# Impacts of priming on distinct immunosuppressive mechanisms of mesenchymal stromal cells under translationally relevant conditions

**DOI:** 10.1186/s13287-024-03677-5

**Published:** 2024-03-05

**Authors:** Nick Herger, Irina Heggli, Tamara Mengis, Jan Devan, Leonardo Arpesella, Florian Brunner, Oliver Distler, Stefan Dudli

**Affiliations:** 1 Center of Experimental Rheumatology, Department of Rheumatology, University Hospital Zurich, University of Zurich, Zurich, Switzerland; 2 Department of Physical Medicine and Rheumatology, Balgrist University Hospital, University of Zurich, Balgrist Campus, Zurich, Switzerland; 3 Department of Microbiology and Immunology, The University of Melbourne, Melbourne, VIC Australia

**Keywords:** Mesenchymal stromal cells, MSC, Priming, 3D culture, Hypoxia, Immunomodulation

## Abstract

**Background:**

The multimodal properties of mesenchymal stromal cells (MSCs), particularly their ability to modulate immune responses is of high interest in translational research. Pro-inflammatory, hypoxic, and 3D culture priming are promising and often used strategies to improve the immunosuppressive potency of MSCs, but the underlying mechanisms are not well understood. Therefore, the aims of this study were (i) to compare the effects of pro-inflammatory, hypoxic, and 3D culture priming on the in vitro immunosuppressive potential of MSCs, (ii) to assess if immunosuppressive priming effects are temporally preserved under standard and translationally relevant culture conditions, and (iii) to investigate if the three priming strategies engage the same immunosuppressive mechanisms.

**Methods:**

Functional in vitro T cell suppressive potency measurements were conducted to assess the impact of pro-inflammatory, hypoxic, and 3D culture priming on the immunosuppressive potential of human bone marrow-derived MSCs. Primed MSCs were either cultured under standard cell culture conditions or translationally relevant culture conditions, and their transcriptomic adaptations were monitored over time. Next-generation sequencing was performed to assess if different priming strategies activate distinct immunosuppressive mechanisms.

**Results:**

(i) Pro-inflammatory, hypoxic, and 3D culture priming induced profound transcriptomic changes in MSCs resulting in a significantly enhanced T cell suppressive potential of pro-inflammatory and 3D culture primed MSCs. (ii) Priming effects rapidly faded under standard cell culture conditions but were partially preserved under translationally relevant conditions. Interestingly, continuous 3D culture priming of MSCs maintained the immunosuppressive potency of MSCs. (iii) Next-generation sequencing revealed that priming strategy-specific differentially expressed genes are involved in the T cell suppressive capacity of MSCs, indicating that different priming strategies engage distinct immunosuppressive mechanisms.

**Conclusion:**

Priming can be a useful approach to improve the immunosuppressive potency of MSCs. However, future studies involving primed MSCs should carefully consider the significant impact of translationally relevant conditions on the preservation of priming effects. Continuous 3D culture could act as a functionalized formulation, supporting the administration of MSC spheroids for a sustainably improved immunosuppressive potency.

**Supplementary Information:**

The online version contains supplementary material available at10.1186/s13287-024-03677-5.

## Introduction

Mesenchymal stromal cells (MSCs) have an exceptional clinical potential due to their multi-lineage differentiation capacity, ease of isolation, engraftment potential, self-renewal capacity, favorable safety profile, and immunomodulatory properties [1]. Over 450 completed interventional clinical trials using MSCs have been listed in the ClinicalTrials.gov database (accessed on 10 August 2023). The fields of application of MSCs encompass a wide range of diseases, including degenerative disorders and chronic inflammatory conditions, cancer, COVID-19, graft-versus-host disease, and the repair of fractures and articular cartilage defects. Despite the large number of completed clinical trials on MSCs, only a handful of MSC-based products made it on the market. The reasons are manifold. Briefly, the potency of MSCs and, consequently, the outcomes of clinical trials is influenced by donor selection, tissue origin, lack of standardized isolation methods and culture protocols, insufficient MSC characterization, the absence of meaningful critical quality attributes, and the administration route [2, 3]. In 2019, the International Society of Cell and Gene Therapy (ISCT) refined their existing definition of MSCs with the goal to address the emerging clinical challenges and to standardize the use of MSCs [4]. In addition to fulfilling the three minimal criteria for MSC definition (plastic adherence, surface marker expression, trilineage differentiation), the ISCT recommended to additionally demonstrate the functional properties of MSCs based on functional assays [5, 6]. 

Priming has been suggested as a promising bioengineering strategy to overcome challenges encountered in MSC therapies. It may enhance several therapeutically relevant properties of MSCs such as immunomodulation, migration, tissue regeneration, survival and engraftment, angiogenesis, and stemness [7]. Pre-conditioning MSCs with pro-inflammatory cytokines or exposing MSCs to hypoxia represent the most commonly used priming strategies to improve the therapeutic potential of MSCs [7–9]. 3D culture priming represents a non-genetic emerging MSC priming strategy that was shown to enhance the immunomodulatory, pro-angiogenic, and regenerative properties of MSCs [10–14]. 

The ability of MSCs to modulate a wide range of immune cells is not only pivotal for their therapeutic promise in treating immune disorders, but it also holds great potential for tissue regeneration. Besides the high cellular plasticity and self-renewal capacity of MSCs, the immunomodulatory capacity is likely a crucial contributor to their regenerative potential [15, 16]. The immunomodulatory potential of MSCs is typically assessed by in vitro T cell suppression assays [17]. Interferon gamma (IFNγ) is the most commonly used pro-inflammatory agent for MSC priming to increase their immunomodulatory potency [7, 11, 15]. The combination of IFNγ with tumor necrosis factor alpha (TNFα) was shown to synergistically improve the therapeutic potential of MSCs [18–21]. The exposure of MSCs to oxygen concentrations as low as 1% O_2_ is a commonly applied priming strategy to activate anti-apoptotic and angiogenic pathways [22, 23]. Recent data demonstrated that hypoxia also enhances the immunomodulatory capacity of MSCs [9, 24]. The aggregation of MSCs into 3D spheroids is an emerging priming strategy, which was shown to enhance the immunomodulatory capacity of MSCs [12]. The immunomodulatory mechanism of 3D culture priming is likely distinct from those of pro-inflammatory and hypoxic priming [25]. 

The main goal of priming is to solve the clinical challenges faced by MSC products to ultimately administer them to patients in need. However, the lack of standardized priming strategies combined with a limited understanding of the effects of priming on MSC function, hinder the translational potential of MSC priming. To consider a priming strategy for clinical application, MSCs must preserve the priming effects post-administration. Little information is available whether MSCs can preserve the priming effects over time. If primed MSCs are administered to patients, they will be exposed to disease-specific microenvironments. The influence of local microenvironments on the temporal preservation of priming effects is not well studied. Therefore, the aims of this study were (i) to investigate the effects of pro-inflammatory, hypoxic, and 3D culture priming on the in vitro immunosuppressive potential of MSCs, (ii) to assess if immunosuppressive priming effects are temporally preserved under standard cell culture conditions, or if translationally relevant conditions are necessary to maintain the priming effects over time, and (iii) to clarify if pro-inflammatory, hypoxic, and 3D culture priming engage the same immunosuppressive mechanisms.

## Materials and methods

### Experimental design

**Fig. 1:**
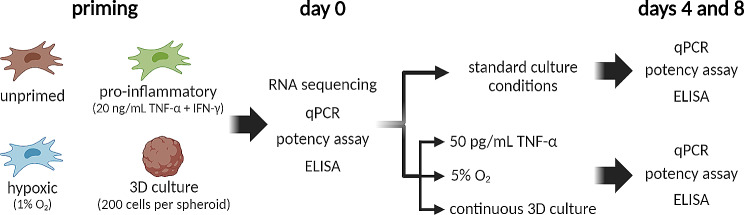
Illustration of the experimental setup. Created with BioRender.com

Commercial human iliac crest-derived bone marrow MSCs from three donors were used for this study. The MSCs were either primed by pro-inflammatory, hypoxic, or 3D culture priming for 48 h (Fig. 1). Unprimed MSCs served as comparator. Following priming, RNA was isolated from a fraction of the MSCs for RNA sequencing and quantitative real-time polymerase chain reaction (qPCR). The remaining MSCs were either used for the in vitro functional immunosuppressive potency assay or cultured under two separate culture conditions (standard conditions or translationally relevant conditions). The translationally relevant conditions were priming approach-specific, i.e. following hypoxic priming, MSCs were cultured in oxygen concentration levels of a possible MSC recipient tissue in vivo (5% O_2_); following pro-inflammatory priming, MSC were cultured in recipient tissue typical inflammation level (50 pg/mL TNF-α); and following 3D culture priming, MSC were kept in spheroids, because this represents the translational scenario of spheroid injection. On days 4 and 8, the primed MSCs were harvested and analyzed by qPCR and with the in vitro immunosuppressive potency assay.

### Preparation of peripheral blood mononuclear cells and expansion of MSCs

The study was conducted in accordance with the declaration of Helsinki and approved by the local ethics commission (ethical approval: BASEC-Nr. 2018 − 01873). Commercial human iliac crest-derived bone marrow MSCs from three donors were used (RoosterBio, Maryland, USA). MSC donor information is detailed in the supplementary Table 1. The MSCs conform with the ISCT minimal criteria for human MSC identity and were between population doubling levels 12 and 14 for all conducted experiments. MSCs were expanded and primed in minimum essential medium alpha (Biowest, Nuaillé, France) supplemented with 5% human platelet lysate (STEMCELL Technologies, Vancouver, Canada) and 50 U/mL penicillin/streptomycin (Life Technologies, California, USA) in a humidified incubator at 37 °C, 21% O_2_, and 5% CO_2_. Media change was performed twice a week. Whole blood was obtained from a single healthy donor and peripheral blood mononuclear cells (PBMCs) were isolated using Lymphoprep (STEMCELL Technologies, Vancouver, Canada) density gradient medium. The PBMCs were subsequently aliquoted and frozen in 10% dimethyl sulfoxide (Carl Roth, Karlsruhe, Germany) in heatinactivated fetal calf serum (Life Technologies, California, USA) for 24 h at -80 °C, in a CoolCell LX cell freezing container (Corning, New York, USA), before being transferred to a liquid nitrogen tank. For the coculture of MSCs with PBMCs, RPMI 1640 (Biowest, Nuaillé, France) supplemented with 10% heatinactivated fetal calf serum, 50 U/mL penicillin/streptomycin (Life Technologies, California, USA), 200 nM Lglutamine (Life Technologies, California, USA), and 2.5 ng/mL recombinant human basic fibroblast growth factor (Peprotech, New Jersey, USA) was used. No media change was performed throughout the co-culture experiment.

### MSC priming and culture

#### Proinflammatory priming

MSCs were treated with a combination of 20 ng/mL TNFα (Peprotech, New Jersey, USA) and 20 ng/mL IFNγ (Peprotech, New Jersey, USA) in the priming medium for 48 h according to a previously published priming protocol [26]. Following pro-inflammatory priming, MSCs were either cultured in standard cell culture medium without any supplemented inflammatory cytokines, or supplemented with 50 pg/mL TNFα to mimic the inflammation level of a possible MSC recipient tissue in vivo [27, 28].

#### Hypoxic priming

MSCs were exposed to 1% O_2_ in a humidified incubator at 37 °C and 5% CO_2_ for 48 h. Following hypoxic priming, MSCs were either cultured in standard cell culture medium under normoxia (21% O_2_) or under 5% O_2_ to simulate the oxygen concentration of possible recipient organs, e.g. bone, brain, heart, and liver [29, 30].

#### 3D culture priming

SP5D plates (Kugelmeiers, Zurich, Switzerland) were used for the 3D culture priming of MSCs (supplementary Fig. 1). 1.5 × 10^5^ MSCs were seeded per SP5D well and incubated for 48 h to generate 750 spheroids each containing approximately 200 MSCs. MSC spheroids were dissociated by digestion in 500 µg collagenase P (Hoffmann LA Roche, Basel, Switzerland) dissolved in 1 mL Hank’s balanced salt solution (Sigma-Aldrich, Missouri, USA) for 1 h at 37 °C, resulting in a single cell suspension. MSCs cultured as spheroids were viable and metabolically active for the duration of culture (supplementary Fig. 2). Following the 48 h 3D culture priming, MSC spheroids were dissociated and cultured in monolayers under standard cell culture conditions (37 °C, 21% O_2_, and 5% CO_2_). To simulate the translational scenario of spheroid injection, the 3D culture primed MSCs were continuously cultured as spheroids.

### In vitro functional immunosuppressive potency assay

Pro-inflammatory and hypoxic primed MSCs were harvested using Accutase (Innovative Cell Technologies, California, USA) and 3D culture primed MSCs were dissociated as described above. 3 × 10^4^ primed MSCs were seeded in Fbottomed 96well plates and cultured for 24 h in coculture medium to allow MSCs to adhere to the plate. PBMCs were thawed, resuspended in coculture medium, and incubated overnight under standard cell culture conditions. The PBMCs were labeled with 2.3 µM CellTrace CFSE Cell Proliferation Kit (Life Technologies, California, USA) in Dulbecco’s phosphate-buffered saline (PBS) (PAN-Biotech, Bayern, Germany) for 5 min at room temperature, washed, and 10^5^ CFSEPBMCs were directly added to the adherent MSCs in the 96well plate. The MSC:PBMC ratio of 30:100 was determined based on a preliminary titration experiment (supplementary Fig. 3). To activate T cells and induce T cell proliferation 1 µL/100 µL TransAct (Miltenyi Biotec, Nordrhein Westfalen, Germany) – a nanomatrix conjugated to recombinant humanized CD3 and CD28 agonists – was directly added to the coculture. The co-culture of MSCs with PBMCs was preferred over the co-culture with isolated T cells to more closely mimic physiological conditions. Additionally, the co-culture of MSCs with PBMCs preserves the natural interactions between MSCs and cell types other than T cells, which allows for the detection of indirect T cell suppressive effects, for example via the modulation of monocytes. MSCs were cocultured with CFSEPBMCs for 3 days in a humidified incubator at 37 °C, 21% O_2_, and 5% CO_2_. CFSEPBMCs were subsequently harvested and processed for flow cytometry analysis. CFSEPBMCs were stained with anti-CD3 PE-Cy5, anti-CD4 BV711, and anti-CD8 PE (all from BioLegend, California, USA) and a LIVE/DEAD fixable viability dye (Life Technologies, California, USA) was used to exclude dead cells from the analysis. CFSEPBMCs were stained for 1 h at room temperature. Stained CFSEPBMCs were washed with PBS and analyzed using a BD LSRFortessa cell analyser (BD Biosciences, New Jersey, USA). Data was analyzed with FlowJo v10.8 software. Cell doublets and dead cells were excluded from the analysis. The proliferation of viable T cells (CD3^+^), helper T cells (CD4^+^), and cytotoxic T cells (CD8^+^) was calculated based on the CFSE fluorescence intensity. T cells cultured in the absence of co-cultured MSCs served as proliferation control. FlowJo’s proliferation modeling tool was utilized for calculating the proliferation index (total number of cell divisions divided by the number of cells that went into cell division). Proliferation index is presented on a scale from 0% (complete suppression of CFSE-PBMC proliferation) to 100% (CFSE-PBMCs proliferation in co-culture with unprimed MSCs). It is important to highlight that a low proliferation index indicates strong immunosuppression.

### RNA sequencing and data analysis

RNA was isolated from unprimed, pro-inflammatory, hypoxic, and 3D culture primed MSCs directly following 48 h priming, using the RNA isolation protocol described below. Library preparation was performed with 500 ng total RNA using the poly(A)-based TruSeq Stranded mRNA kit (Illumina, California, USA). Libraries were sequenced using the NovaSeq 6000 (Illumina, California, USA) sequencing system (16 million reads per sample). The quality of the data was assessed using FastQC and the readings were mapped to the reference genome hg38 using STAR v.2.7.10b. Genes of primed MSCs with FDR ≤ 0.1 and |log_2_ fold change| ≥ 1.5 (log_2_FC) relative to the gene expression of unprimed MSCs were considered differentially expressed. Principle component analysis (PCA) was calculated based on the top 2000 genes ranked by standard deviation. A Venn diagram of significantly differentially expressed genes (DEGs) of primed versus unprimed MSCs was generated. For the volcano plots, DEGs of primed MSCs versus unprimed MSCs were ranked from highest |log_2_FC| values to lowest. Afterwards, the list was filtered by all genes that were part of GO:0006955 (immune response) or GO:0006954 (inflammatory response). The top 10 DEGs from the resulting list were identified and highlighted in the volcano plots and listed in the supplementary Table 2. Hypergeometric over-representation analyses (ORA) were performed separately for upregulated and downregulated DEGs using the R package clusterProfiler v.3.17. Top 5 over-represented biological processes were selected based on adjusted p-values.

### Quantitative real-time polymerase chain reaction

A fraction of primed MSCs was resuspended in buffer RLT (Qiagen, Venlo, Netherlands) supplemented with 10 µL/mL 2mercaptoethanol (Merck, Darmstadt, Germany) for RNA isolation. RNA was isolated from MSCs using the RNeasy Mini Kit (Qiagen, Venlo, Netherlands) and cDNA was synthesized using SensiFAST cDNA Synthesis Kit (Solis BioDyne, Tartu, Estonia) according to the manufacturer’s instructions. qPCR was performed in 384 white well qPCR plates on a CFX Opus Real-Time qPCR System (Bio-Rad, California, USA) using the HOT FIREPol EvaGreen qPCR Supermix (Solis BioDyne, Tartu, Estonia) using primers listed in supplementary Table 3. We used a qPCR reaction volume of 10 µL and the following cycling protocol: 95 °C for 12 min followed by 45 cycles performed at 95 °C for 15 s, 65 °C for 30 s, and 72 °C for 30 s. Relative fold gene expressions were calculated using the 2^−∆∆Cq^ method and TATA-box binding protein (TBP) served as housekeeping gene.

### Enzyme-linked immunosorbent assay (ELISA)

Supernatants of MSC cultures were collected at days 0, 4, and 8. Media changes were performed at days 2 and 6 to ensure that each collected supernatant was conditioned for 48 h. The concentration of PGE2, CXCL9, and kynurenine were quantified in undiluted supernatants and their concentration per 10^5^ MSCs was calculated. For each priming strategy, one representative protein was selected, which demonstrated strong upregulation immediately following priming on the transcriptomic level. PGE_2_ and CXCL9 ELISA kits were acquired from Abcam (Cambridge, United Kingdom), the kynurenine EILSA kit was acquired from AssayGenie (Dublin, Ireland).

### Selection of signature genes

Three gene sets were assembled to reflect the immunomodulatory mechanisms of pro-inflammatory, hypoxic, and 3D culture primed MSCs. Each gene set contained five immunomodulation-associated signature genes (DEGs of GO:0006955 or GO:0006954) that were significantly upregulated after respective priming. The selection of the signature genes was based on our RNA sequencing data of primed versus unprimed MSCs and has been cross-referenced with immunomodulatory potency markers published in literature [31–39]. Only DEGs and immunomodulation-associated genes were considered. A fourth gene set was created containing five immunomodulatory potency markers, which are frequently used in literature [40–44]. This gene set was investigated in all primed MSCs, regardless of which priming strategy was used. We quantified the signature genes using qPCR to evaluate the temporal stability of different priming approaches and to study how translationally relevant microenvironmental factors influence the priming effects over time. To test if the immunomodulation-associated genes correlate with T cell suppression, we quantified all twenty signature genes (5 pro-inflammatory, 5 hypoxic, 5 3D culture, and 5 frequently used) for pro-inflammatory, hypoxic, and 3D culture primed MSCs. We hypothesized that different priming mechanisms engage different immunomodulatory mechanisms and hence correlate to varying degrees with T cell suppression.

### Statistical analysis

Statistical analysis was performed with GraphPad PRISM v.10.0.1. Significance level was α = 0.05, if not stated otherwise. To compare the effects of priming, time, and culture condition on T-cell suppression, a three-way ANOVA was calculated followed by Tukey post-hoc test corrected for multiple testing using Bonferroni p-value adjustment. To investigate which priming strategy (represented by the signature genes) shows best correlation with T cell suppression, Pearson correlations between the expression levels of signature genes and T cell suppression were computed. Priming strategies with strongest correlations are expected to have the highest T cell suppressive effects.

## Results

First, we conducted an immune functional potency assay on primed and unprimed MSCs. Our main goal was to evaluate if pro-inflammatory, hypoxic, and 3D culture priming can enhance the in vitro functional immunosuppressive potency of MSCs. Second, we assessed if the in vitro functional immunosuppressive potencies of differently primed MSCs are stable over time. We hypothesized that priming effects fade over time but can be partially preserved by translationally relevant conditions i.e. pro-inflammatory environment, hypoxic environment, and continuous 3D culture. Third, we tested if the three priming strategies engage the same immunosuppressive mechanisms.

### Pro-inflammatory and 3D culture priming enhance the T cell suppressive capacity of MSCs

**Fig. 2:**
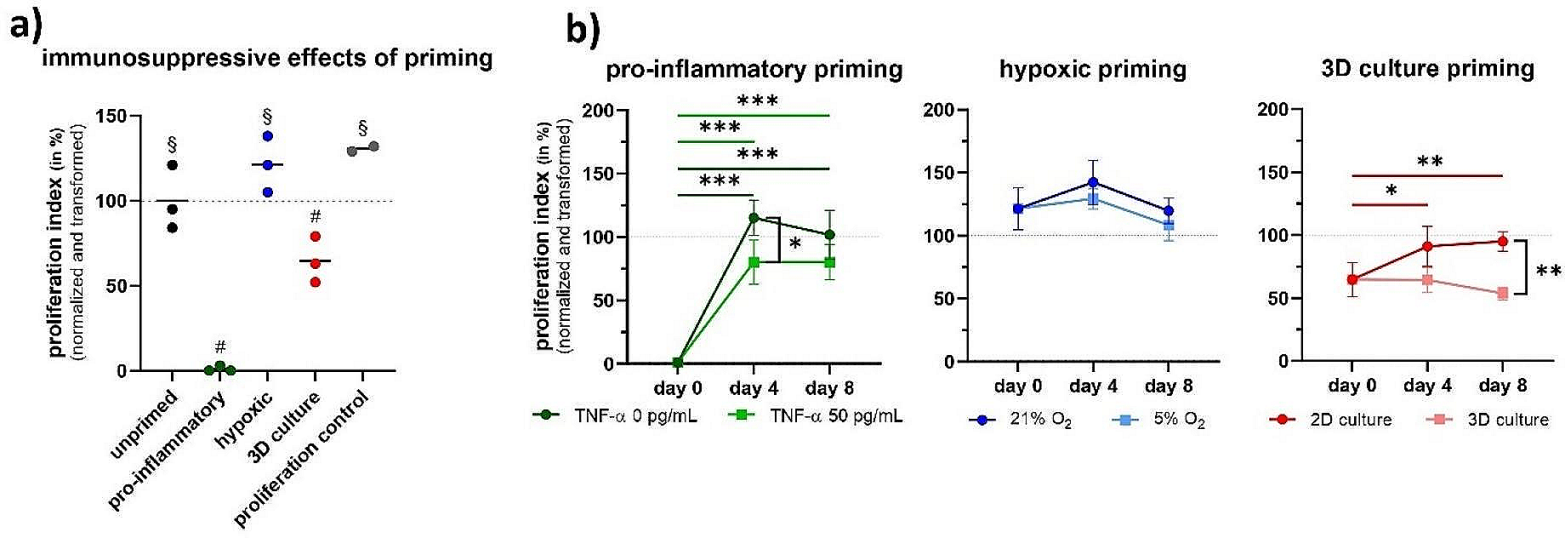
The effects of each priming strategy on the immunosuppressive potential of MSCs were measured by an in vitro functional potency assay following 48 h of priming. A normalized transformed proliferation index of 0% corresponds to the complete suppression of CD3^+^ T cell proliferation. A value of 100% indicates that the T cells proliferated equivalent to T cells co-cultured with unprimed MSCs (grey dashed horizontal lines). (**a**) Different priming strategies enhance the T cell suppressive capacity of MSCs. (**b**) Priming effects fade over time but are partially preserved by translationally relevant conditions. The proliferation control represents the proliferation of T cells in the absence of co-cultured MSCs. Data points represent averages ± standard deviations of the MSCs from three healthy donors. # indicate significant differences (*p* < 0.001, except unprimed vs. 3D culture primed *p* = 0.03) against all other conditions. § indicate significant differences (*p* < 0.001) against pro-inflammatory and 3D culture priming. *: *p* ≤ 0.05; **: *p* ≤ 0.01; ***: *p* ≤ 0.001

Differently primed MSCs have different in vitro T cell suppressive potencies (*p* < 0.001, Fig. 2a). The T cell suppressive capacities of differently primed MSCs were compared to unprimed MSCs. Pro-inflammatory priming significantly enhanced the immunosuppressive capacity of MSCs compared to unprimed, hypoxic primed, and 3D culture primed MSCs, directly following 48 h of priming. Pro-inflammatory primed MSCs achieved a complete suppression of T cell proliferation (mean difference ± standard error = -99% ± 11%, *p* < 0.001). Hypoxic priming had no beneficial effect on the T cell suppressive potential of MSCs (mean difference ± standard error = + 21% ± 11%, *p* > 0.48). 3D culture priming significantly increased the immunosuppressive capacity of MSCs (mean difference ± standard error = -35% ± 11%, *p* = 0.03), but to a lesser extent than pro-inflammatory priming (*p* < 0.001).

### Priming effects fade over time but are partially preserved by translationally relevant conditions

Priming effects were not stable over time under standard cell culture condition (*p* < 0.001, Fig. 2b). The strongly enhanced immunosuppressive capacity of pro-inflammatory primed MSCs relative to unprimed MSCs entirely disappeared already four days after priming (mean difference ± standard error for days 4 and 8: +15% ± 11% (*p* = 0.84), + 2% ± 11% (*p* > 0.99)). 3D culture primed MSC lost their significantly enhanced immunosuppressive capacity as well at later time points (mean difference ± standard error for days 4 and 8: -9% ± 11% (*p* > 0.99), -5% ± 11% (*p* > 0.99)). Hypoxic primed MSCs even favored T cell proliferation at day 4 (mean difference ± standard error: +42% ± 11% *p* = 0.005).

Translationally relevant culture conditions had a significant impact on the temporal preservation of priming effects (*p* < 0.001, Fig. 2b). The T cell suppressive capacities of MSCs cultured under translationally relevant conditions were compared to the T cell suppressive conditions of MSC cultured under standard cell culture conditions. A continuous stimulation of pro-inflammatory primed MSCs with as little as 50 pg/mL TNFα was enough to partially preserve immunosuppressive potency up to four days (mean difference ± standard error for days 4 and 8: -35% ± 11% (*p* = 0.04), -22% ± 11% (*p* = 0.46)). No notable effect of translationally relevant 5% O_2_ was found (mean difference ± standard error for days 4 and 8: -13% ± 11% (*p* = 0.92), -11% ± 11% (*p* = 0.96)). Continuous spheroid culture completely rescued the enhanced immunosuppressive priming effects from fading (mean difference ± standard error for days 4 and 8: -27% ± 11% (*p* = 0.21), -41% ± 11% (*p* = 0.007)). We observed no noticeable differences regarding the potency of MSCs to suppress total T cells, Thelper cells, and cytotoxic T cells (supplementary Figs. 4 and 5).

### Different priming strategies induce distinct cellular mechanisms

**Fig. 3:**
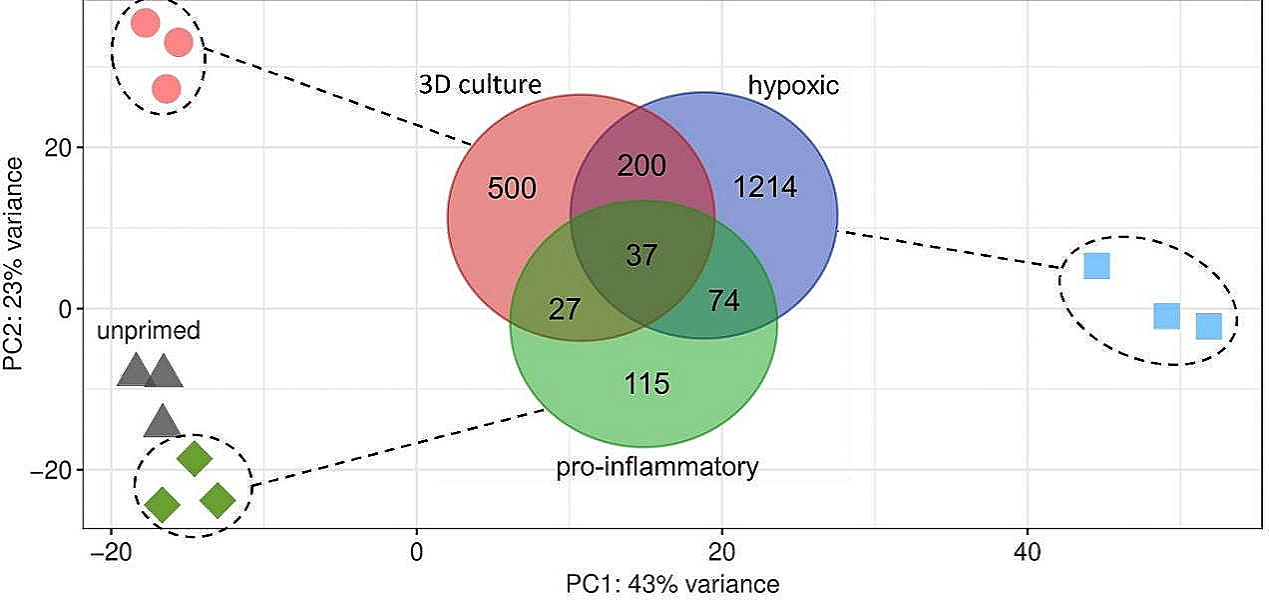
The PCA plot revealed a distinct clustering of pro-inflammatory, hypoxic, 3D culture primed, as well as unprimed MSCs from three donors. Notably, the MSCs clearly cluster by priming strategy and not by donors. The top 2000 genes ranked by standard deviation were considered for this PCA plot. The Venn diagram shows for each priming strategy, the numbers of significant DEGs (FDR ≤ 0.1, |log_2_ ratio| ≥ 1.5) compared to unprimed MSCs. Additionally, the Venn diagram highlights the numbers of overlapping DEGs between priming strategies to indicate similarities or dissimilarities between different priming strategies

In order to evaluate if pro-inflammatory, hypoxic, and 3D culture priming engage the same immunosuppressive mechanisms, we performed RNA sequencing after priming. We observed largely different effects of the three priming strategies on the transcriptome of MSCs as evidenced by the distinct clustering of the priming strategies in the PCA plot (Fig. 3). Hypoxic priming induced the largest transcriptomic changes, whereas pro-inflammatory primed MSCs clustered closest to unprimed MSCs. The distinct clustering of the differently primed MSCs in the PCA plot is also reflected by the individual numbers of DEGs compared to unprimed MSCs, as shown in the Venn diagram. Several genes were differentially expressed in more than one priming strategy, suggesting possible similarities between the priming strategies (supplementary Tables 4, 5, 6, 7).

**Fig. 4:**
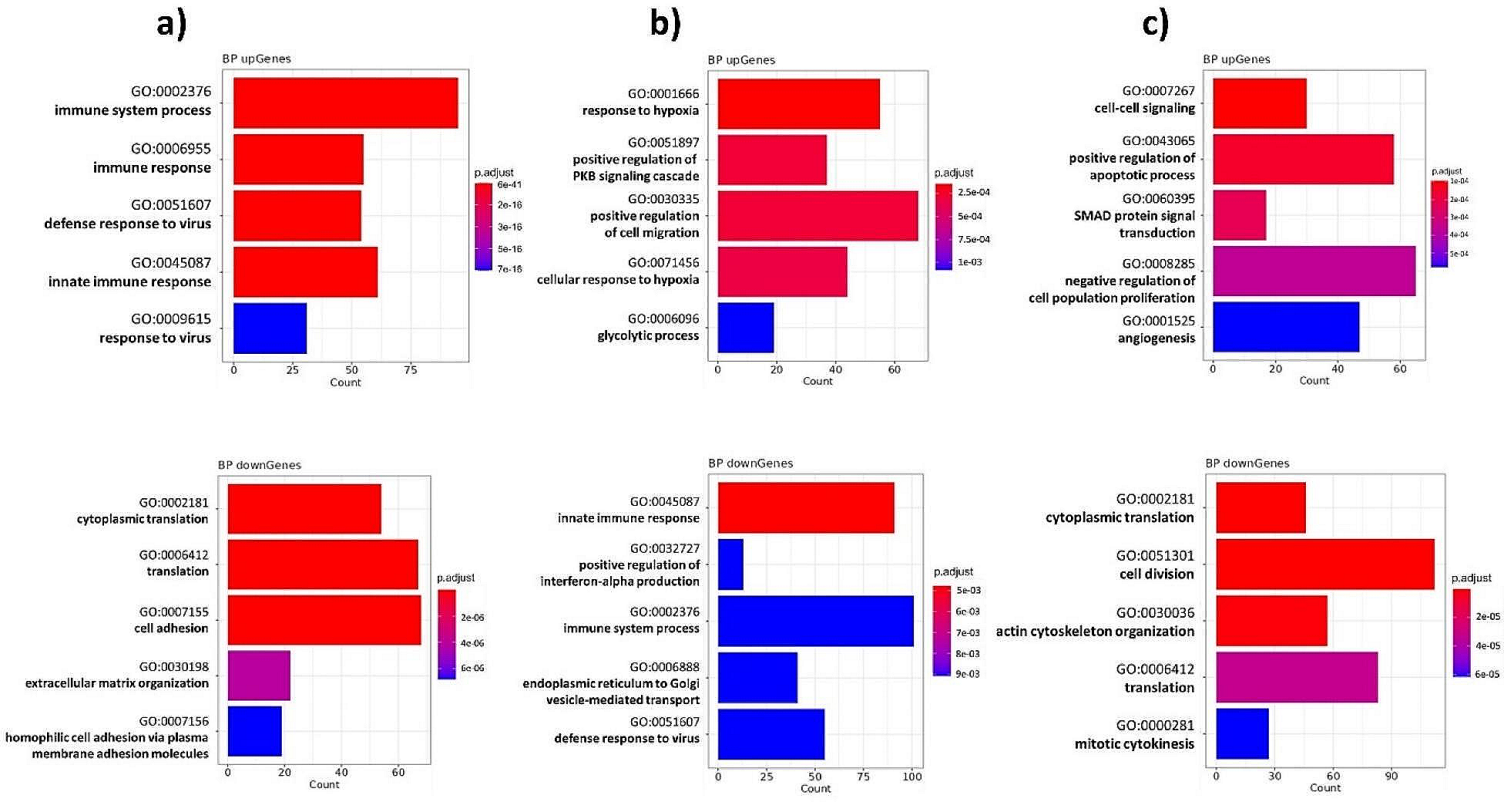
The bar plots show the top five upregulated and top five downregulated biological processes of (**a**) pro-inflammatory, (**b**) hypoxic, or (**c**) 3D culture primed MSCs compared to unprimed MSCs. The upper bar plots reflect the upregulated biological processes, and the lower bar plot represents the downregulated biological processes. Only enriched biological processes based on DEGs with p-values ≤ 0.01 are shown

To better understand the transcriptomic changes induced by priming, we performed an ORA with the DEGs to identify the underlying biological processes (Fig. 4).

Pro-inflammatory priming led to the upregulation of genes involved in immune system responses and defense response to virus. In contrast, genes associated with protein synthesis, cell adhesion, and extracellular matrix organization were significantly downregulated. Hypoxic priming mainly induced adaptations to an anaerobic metabolism as well as an increased cellular migration potential. In contrast to pro-inflammatory priming the transcriptome of hypoxic primed MSCs revealed a downregulation of genes involved in immune system responses, corroborating findings from T cell suppression. MSCs cultured as spheroids exhibited substantial transcriptomic changes involving the cytoskeleton and cellular signaling. Furthermore, cell division was downregulated likely due to contact-inhibition, while apoptotic and angiogenic processes were upregulated. It is well established that MSCs cultured in self-aggregated spheroids form necrotic cores, due to limited nutrient availability at the core of spheroids [45]. Our small MSC spheroids (200 MSCs per spheroid, approximately 120 μm diameter) had few apoptotic cells and almost no necrotic cells (supplementary Fig. 2). Yet, upregulation of angiogenic processes could be a possible reaction to the oxygen and nutrient deprived spheroid core.

**Fig. 5:**
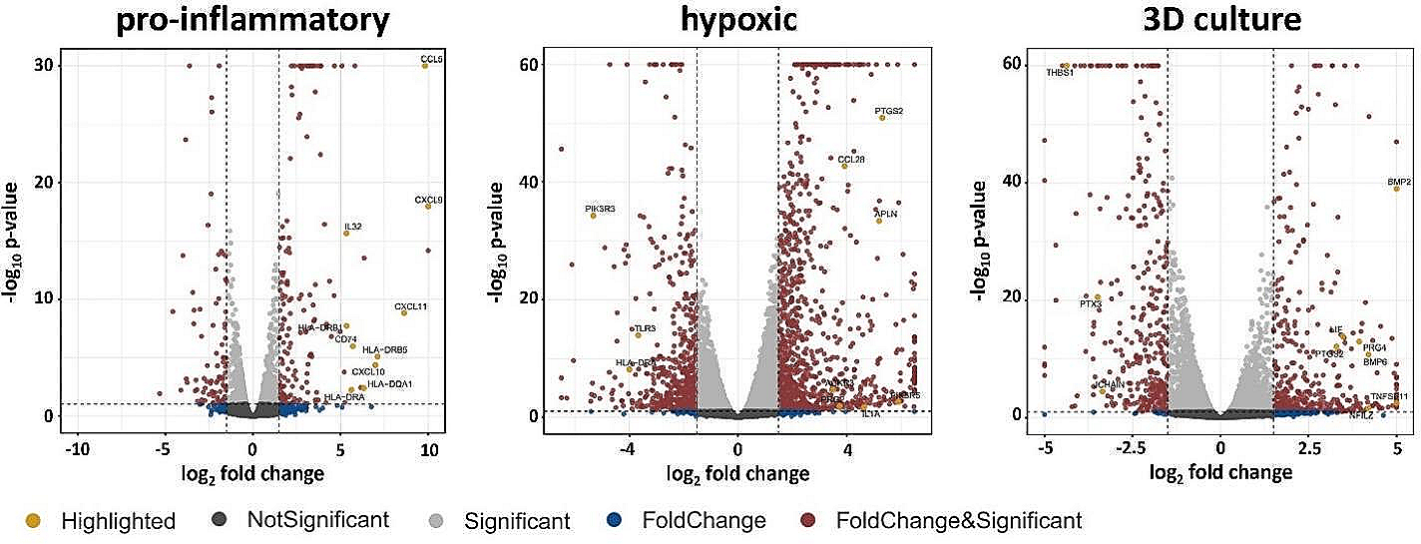
The volcano plots show the p-values and fold changes of the DEG analysis of primed versus unprimed MSCs. The horizontal dotted line represents the p-value threshold of 0.05 and the two vertical dotted lines represent the log_2_FC thresholds of ± 1.5. The DEGs were ranked from highest |log_2_FC| values to lowest and the top 10 immune-response related DEGs were identified for each priming strategy separately. DEGs that were part of either GO:0006955 (immune response) or GO:0006954 (inflammatory response) were considered immune-response related and the top 10 immune-response related DEGs were highlighted in the volcano plots

To investigate if differently primed MSCs engage distinct immunomodulatory mechanisms, we first identified DEGs involved in immunomodulation. DEGs that were either part of GO:0006955 (immune response) or GO:0006954 (inflammatory response) were considered immunomodulation-associated. The two selected gene ontologies represent high-level gene ontologies encompassing the majority of sub-ontologies involved in immunomodulation. The top 10 immunomodulation-associated DEGs were identified for each priming strategy separately (supplementary Table 2) and highlighted in volcano plots (Fig. 5). 19% (49/253) of DEGs in pro-inflammatory primed MSCs were immunomodulation-associated. This fraction was lower in hypoxic (5%, 69/1525) and 3D culture (6%, 45/764) primed MSCs. In pro-inflammatory primed MSCs, all 10 immunomodulation-associated DEGs were upregulated. In hypoxic and 3D culture primed MSC, 7 were up- and 3 were down-regulated. None of the top 10 immunomodulation-related DEGs were shared between all three priming strategies and only two were shared between two priming strategies, suggesting that differently primed MSCs engage distinct immunomodulatory mechanisms. Prostaglandin-endoperoxide synthase 2 (*PTGS2)* was strongly upregulated in hypoxic primed and 3D culture primed MSCs. Major histocompatibility complex class II DR alpha *(HLADRA)* was upregulated in pro-inflammatory primed MSCs but downregulated in hypoxic primed MSCs. This aligns with the observations from the ORA where pro-inflammatory priming and hypoxic priming showed opposing immune system response-related transcriptional changes. The top 10 immunomodulation-associated DEG of pro-inflammatory primed MSC were mainly involved in immune cell trafficking, antigen presentation, and immune response activation. The immunomodulatory DEGs of hypoxic primed MSCs have diverse roles in inflammatory responses ranging from pathogen recognition and presentation to immune cell attraction and migration. Interestingly, several of the top 10 immunomodulation-associated DEGs of 3D culture primed MSCs are not only involved in the modulation of an immune response but also in cell differentiation.

In summary, PCA revealed distinct clustering of the different priming strategies. This indicates specific effects of the priming strategies on the transcriptome of MSCs. Different priming strategies activated separate biological processes as evidenced by the ORA. Most of the top 10 immunomodulation-associated DEGs were unique to the priming strategies. These findings suggest that differentially primed MSCs engage distinct immunomodulatory mechanisms.

### The transcriptomic changes of primed MSCs fade rapidly but can be partially preserved by translationally relevant conditions

**Fig. 6:**
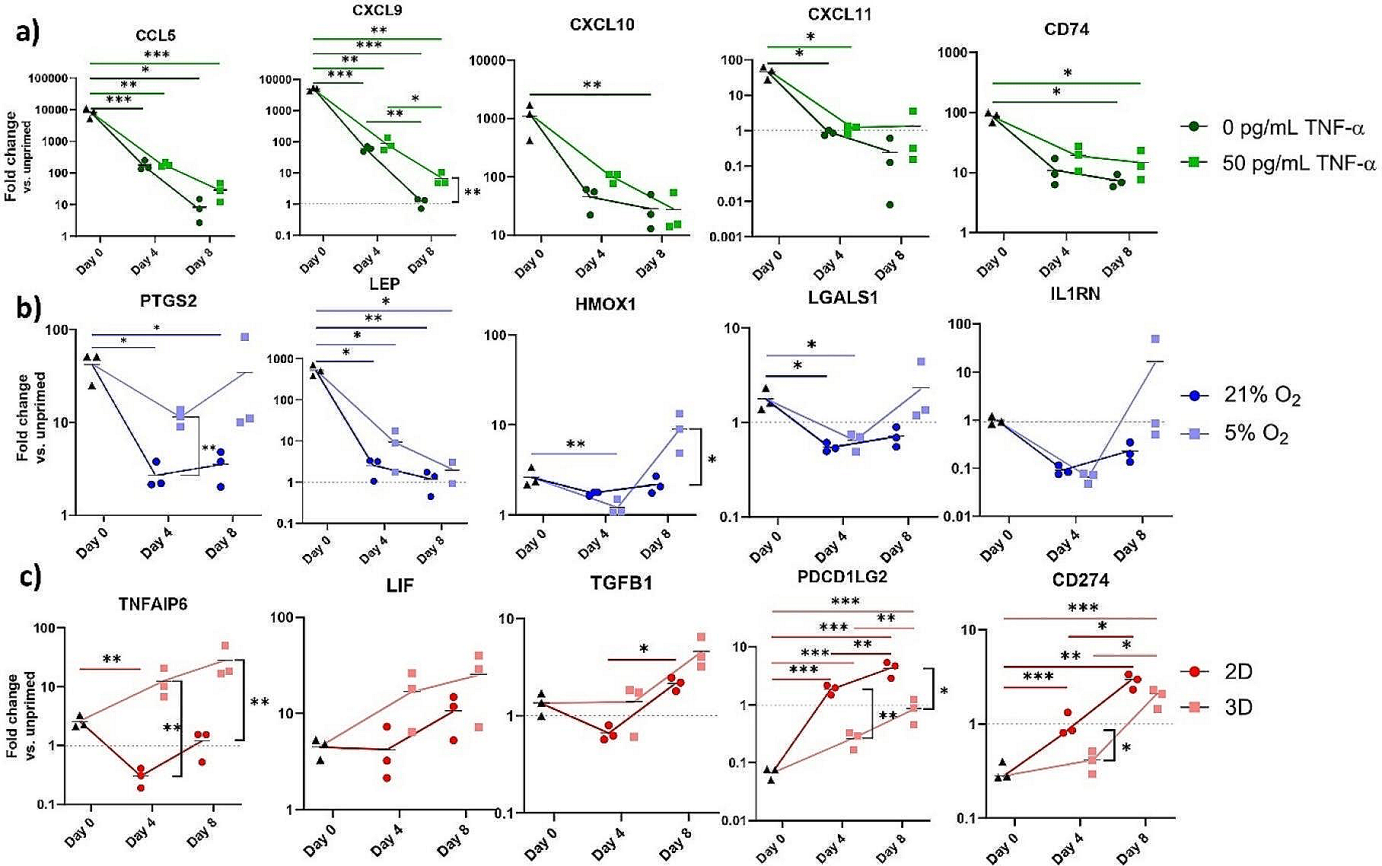
The effects of pro-inflammatory (**a**), hypoxic (**b**), and 3D culture priming (**c**)on the transcriptome of MSCs were semi-quantified by qPCR at three time points: day 0 (directly following 48 h of priming), day 4, and day 8. Fold changes are shown and represent the gene expression levels of primed MSCs from three healthy donors compared to the gene expression levels of unprimed MSCs at time point day 0. Lines connect the mean fold changes of the three healthy donors. The significance levels for the gene expression differences between each time point and condition, compared to the gene expression directly after priming at day 0 are shown. Additionally, for each time point separately, significant gene expression differences between primed MSCs cultured under standard conditions and primed MSCs cultured under translationally relevant conditions are highlighted by significance indicators (not significant: *p* > 0.05; *: *p* ≤ 0.05; **: *p* ≤ 0.01; ***: *p* ≤ 0.001)

The expression levels of the immunomodulatory signature genes of pro-inflammatory, hypoxic, and 3D culture primed MSCs were analyzed at day 0, 4, and 8 to evaluate the temporal stability of different priming approaches and to study how translationally relevant microenvironmental factors influence the priming effects over time (Fig. 6). The pro-inflammatory priming effect was consistently strongest directly after priming at timepoint day 0. However, the expression levels of all signature genes rapidly faded, and reached expression levels comparable to those of unprimed MSCs. Fading of the pro-inflammatory priming effect was not significantly rescued by the continuous inflammatory stimulation but showed a trend towards improved temporal preservation. The expression levels of the immunomodulatory signature of hypoxic primed MSCs demonstrated moderate fold changes compared to unprimed MSCs. *PTGS2* and leptin (*LEP*) were notably upregulated directly following priming but rapidly faded under normoxic conditions. Physiological oxygen concentrations partially preserve the expression levels of multiple signature genes. Surprisingly, despite the significant differential expression of the 3D culture priming-specific signature genes in RNA sequencing, qPCR revealed weak differential expressions of these genes in 3D culture primed MSCs. Interestingly, when MSCs were continuously cultured in spheroids the gene expression levels of chemotactic factors and immunoregulatory secretory proteins further increased over time. This was not observed for MSCs that were dissociated after 3D culture priming and subsequently cultured as monolayers. We observed comparable outcomes for the frequently used priming strategy-independent immunomodulatory potency markers as shown in supplementary Fig. 6. In general, the priming effects rapidly faded over time, but they were partially preserved under translationally relevant conditions, in particular under continuous 3D culture.

**Fig. 7:**
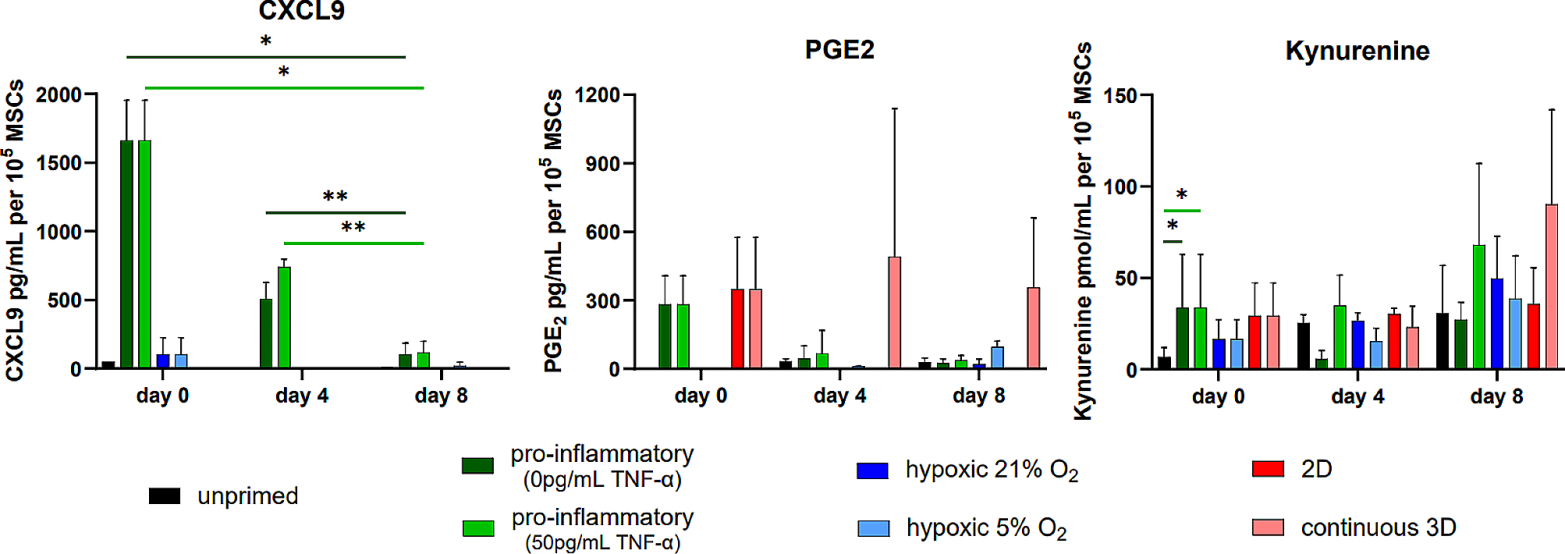
The effects of different priming strategies and culture conditions on the temporal stability of selected proteins in the supernatants of MSC cultures. Proteins were quantified in the supernatants of MSC cultures using ELISA. Supernatants were collected after 48 h of culture with MSCs from three healthy donors at day 0, day 4, and day 8. Media changes were performed at days 2 and 6. Bars represent averages ± standard deviations of the quantified proteins. Not significant: *p* > 0.05; *: *p* ≤ 0.05; **: *p* ≤ 0.01

The rapid fading of priming effects extended beyond the transcriptomic level, as evidenced by declining CXCL9 concentrations in the supernatants of pro-inflammatory primed MSC cultures (Fig. 7). Furthermore, the concentrations of PGE2 and kynurenine – indicative of IDO1 activity – mirrored the gene expression patterns.

### The T cell suppressive mechanism of 3D culture primed MSCs is not reflected by the immunomodulatory potency markers

To identify potentially different T cell suppressive mechanisms, the expression levels of all signature genes (5 pro-inflammatory, 5 hypoxic, 5 3D culture, and 5 literature-based genes) were correlated with the corresponding T cell suppressive potentials. Fourteen genes had a correlation coefficient |r| ≥ 0.5 (Table 1). 11 of them correlated with the pro-inflammatory priming effect, suggesting that the T cell suppression of pro-inflammatory primed MSCs is mediated by the signature genes. Only one gene correlated with T cell suppression of 3D culture primed MSC. This indicates that the T cell suppressive mechanism of 3D culture primed MSCs involves genes and mechanisms different from the gene ontologies GO:0006955 (immune response) and GO:0006954 (inflammatory response).

**Table 1:** Correlation for each priming strategy separately

gene	r	p-value	p/h/3D
*CD74*	-0.93	< 0.001	p
*CXCL9*	-0.90	< 0.001	p
*IDO1*	-0.88	< 0.001	p
*ICAM1*	-0.87	< 0.001	p
*CXCL11*	-0.86	< 0.001	p
*CCL5*	-0.86	< 0.001	p
*TNFAIP6*	-0.80	< 0.001	p
*IL1RN*	-0.80	< 0.001	p
*CXCL10*	-0.80	< 0.001	p
*CXCL8*	-0.77	< 0.001	p
*PDCD1LG2*	+ 0.72	0.002	3D
*LGALS1*	-0.59	0.02	p
*CCL2*	-0.57	0.03	h
*PDCD1LG2*	-0.51	0.05	h

Sorted by descending |r|. CCL5 (CC motif chemokine ligand 5), CXCL9 (CXC motif chemokine ligand 9), CXCL10 (CXC motif chemokine ligand 10), CXCL11 (CXC motif chemokine ligand 11), CXCL8 (CXC motif chemokine ligand 8), CD74 (major histocompatibility complex class II invariant chain), IL1RN (interleukin 1 receptor antagonist), PDCD1LG2 (programmed cell death 1 ligand 2), IDO1 (indoleamine 2,3diogygenase 1), CCL2 (CC motif chemokine ligand 2), TNFAIP6 (TNF alpha induced protein 6), LGALS1 (galectin 1), ICAM1 (intercellular adhesion molecule 1). p: pro-inflammatory priming, h: hypoxic priming, 3D: 3D culture priming

## Discussion

Priming holds great potential in overcoming clinical challenges faced by MSC therapies. Nevertheless, the absence of standardized priming protocols along with a limited understanding of the cellular adaptations to priming represent serious translational hurdles. A clear understanding of the effects of different priming strategies on MSC function is key for the selection of an appropriate priming approach. Importantly, the influence of translationally relevant in vivo environments on the preservation of priming effects likely impacts treatment efficacy and hence, should be carefully considered. Therefore, the aims of our study were (i) to evaluate if pro-inflammatory, hypoxic, and 3D culture priming can enhance the in vitro functional immunosuppressive potency of MSCs, (ii) to assess if the in vitro functional immunosuppressive potencies of differently primed MSCs are temporally preserved under standard and translationally relevant culture conditions, and (iii) to clarify if the three priming strategies engage the same immunosuppressive mechanisms.

First, pro-inflammatory and 3D culture priming significantly enhanced the in vitro T cell suppressive properties of MSCs. Conversely, hypoxic priming led to a notable loss of the inherent T cell suppressive capacity of unprimed MSCs. Therefore, our data showed that different priming strategies have distinct effects on the immunosuppressive potentials of MSCs. Interestingly, a previous study found that hypoxic priming improves the in vitro immunosuppressive capacity of MSCs [9]. A possible explanation for these contradictory results could be the differences in MSC tissue source and passage number as well as differences in the in vitro immunosuppressive potency assay setup. This highlights the need for a standardized approach to assess MSC potency. Efforts towards standardized in vitro potency assays are ongoing but final assessments of the correlation between the in vitro measured immunosuppressive capacity and in vivo clinical efficacy are pending [17]. We measured the functional immunosuppressive potency of MSCs based on their ability to suppress the proliferation of activated T cells. This approach is widely used but only captures a fraction of the immunomodulatory capacity of MSCs, as it was shown that MSCs have the ability to modulate a wide range of immune cells including macrophages, B cells, T cells, natural killer cells, mast cells, neutrophils, dendritic cells, and likely additional cell types [15, 16]. While, assays to measure the immunomodulatory potential of MSCs on cell types other than T cells have been reported in literature, few publications provide detailed assay protocols [42, 46]. Additionally, the pivotal role of T cells in various chronic inflammatory diseases including rheumatoid arthritis, inflammatory bowel disease, and multiple sclerosis is undisputed [47–49]. Therefore, we focused on the frequently assessed ability of MSCs to suppress the proliferation of activated T cells.

Second, priming effects fade over time but are partially rescued from fading by translationally relevant culture conditions. To consider a priming strategy for clinical applications, MSCs must preserve the priming effect after administration to exert their enhanced therapeutic functions. Only few studies investigated the temporal stability of priming effects and discussed the relevance of temporal stability for clinical translation [2, 50]. Pro-inflammatory, hypoxic, and 3D culture priming induced transient cellular adaptations under standard cell culture conditions, as shown by monitoring priming signature genes over time. All priming approaches induced substantial transcriptomic changes in MSCs directly following priming, but these changes rapidly faded only a few days after the priming. The rapid fading of the priming effects extended beyond the transcriptomic level, as evidenced by declining CXCL9 concentrations in the supernatants of pro-inflammatory primed MSC cultures. The fading of the priming effects was accompanied by the loss of enhanced in vitro functional immunosuppressive potentials of both pro-inflammatory and 3D culture primed MSCs. This signifies that MSC therapies utilizing pro-inflammatory or 3D culture priming to enhance the immunosuppressive potency of MSCs should minimize the time between priming and injection to maximize their therapeutic effects.

It is well established that MSCs have a cellular plasticity, and that their functions are tightly regulated by their surrounding microenvironment [18]. However, the influence of translationally relevant factors such as inflammation, hypoxia, and 3D tissue environments on the preservation of priming effects is notably understudied. As microenvironmental factors might alter priming effects and thereby affect MSC potency, we investigated the effects of translationally relevant in vivo conditions on the temporal preservation of priming effects. To maintain the changes introduced by priming and to avoid superimposing other immunosuppressive mechanisms, the translationally relevant culture conditions were aligned with the individual priming strategies. Hence, pro-inflammatory primed MSCs were exposed to pathologic inflammatory TNFα levels, hypoxic primed MSCs were cultured under physiological O_2_ concentrations, and 3D culture primed MSCs were continuously cultured as spheroids to simulate the translational scenario of spheroid injection. Our data show that a weak continuous inflammatory stimulation of 50 pg/mL TNFα was sufficient to demonstrate a trend towards decelerating the fading of the pro-inflammatory priming effect, both on the transcriptomic and functional levels. We selected 50 pg/mL TNFα for the continuous inflammatory stimulation based on the lower limit of reported TNFα concentrations in the synovial fluid of patients with rheumatoid arthritis [27, 28]. Higher concentrations of TNFα or the combination with IFNγ may have preserved the pro-inflammatory priming effect even better. Nevertheless, TNFα concentrations as low as 50 pg/mL demonstrated a promising trend towards partially preserving pro-inflammatory priming effects. This suggests that the administration of pro-inflammatory primed MSCs into an inflamed target tissue could preserve the priming effect and enhanced therapeutic potency. Priming MSCs for 48 h at 1% O_2_ induced substantial transcriptomic adaptations to the low O_2_ levels, including a reduced responsiveness to oxidative stress. Therefore, hypoxic priming may prepare MSCs for O_2_ partial pressures present in frequently targeted tissues, consequently improving cell survival post-administration [29, 30]. Our study shows that the exposure of hypoxic primed MSCs to a translationally relevant O_2_ concentration better preserves the expression of signature genes compared to hypoxic primed MSCs exposed to an atmospheric O_2_ concentration. It could be hypothesized that hypoxic priming pre-administration might be irrelevant, as unprimed MSCs are likely to undergo hypoxic priming following administration into the hypoxic tissue. Further research is needed to conclusively determine whether the in vitro adaptation to hypoxic conditions is necessary to improve cell survival post-administration. Few days following 3D culture priming, MSCs cultured in monolayers lost their significantly enhanced immunosuppressive capacity. In contrast, when MSCs were continuously cultured in spheroids the gene expression levels of chemotactic factors and immunoregulatory secretory proteins further increased over time and fully preserved the enhanced T cell suppressive potential. The signature genes of pro-inflammatory and hypoxic primed MSCs experienced temporal fading, even under translationally relevant conditions. The continuous exposure of MSCs to high pro-inflammatory cytokine concentrations or low oxygen concentrations, as used during priming, would not have reflected translationally relevant conditions, and would potentially have affected cell viability. Complete preservation of the priming effect was a unique property of 3D culture priming. Maintaining MSCs in a continuous 3D culture could act as a functionalized formulation, supporting the administration of MSC spheroids for a sustainably improved immunosuppressive potency, independent of microenvironmental factors necessary to preserve the priming effect.

Third, different priming strategies engage distinct immunosuppressive mechanisms. We observed significant differences in the T cell suppressive potential of differently primed MSCs. While pro-inflammatory and 3D culture priming significantly enhanced the T cell suppressive potential of MSCs, hypoxic primed MSCs had no beneficial effect. These findings can be partially explained by the distinct transcriptomes of the differently primed MSCs. Pro-inflammatory priming clearly led to an upregulation of genes involved in immune system responses.

Hypoxic priming mainly led to adaptations to an anaerobic metabolism and increased the cellular migration potential. In contrast to pro-inflammatory priming, DEGs involved in immune system responses were clearly downregulated in hypoxic primed MSCs and showed an opposing immunomodulation-associated DEGs expression pattern. This aligned with the outcomes of the in vitro immunosuppressive potency measurements. According to literature, cell survival after in vivo administration might benefit from hypoxic priming [7]. Indeed, the transcriptome of hypoxic primed MSCs revealed a reduced response to oxidative stress, which might improve cell survival post-administration.

While pro-inflammatory and 3D culture primed MSCs significantly enhanced in vitro T cell suppressive capacity, albeit not to the same extent, they likely operate through separate immunosuppressive mechanisms. The transcriptomic changes induced by 3D culture priming were substantially different from those caused by pro-inflammatory priming. 3D culture primed MSCs mainly experienced changes to the cytoskeleton, cellular signaling, and the cell cycle. Our RNA sequencing data revealed that none of the top 10 immunomodulation-associated DEGs was shared between 3D culture primed and pro-inflammatory primed MSCs, indicating the activation of separate immunomodulatory mechanisms. Importantly, while the expression levels of multiple signature genes showed strong correlations with the T cell suppressive capacity of pro-inflammatory primed MSCs, the expression levels of the same signature genes demonstrated no significant correlation with the T cell suppressive potential of 3D culture primed MSCs. Surprisingly, 3D culture primed MSCs demonstrated a strong positive correlation between the gene expression levels of *PDCD1LG2* and the proliferation index, suggesting that 3D culture primed MSCs with lower *PDCD1LG2* expression levels are likely to have a higher immunosuppressive capacity. This finding was unexpected as the binding of PDL2 (encoded by the *PDCD1LG2* gene) to programmed cell death protein 1 (PD-1) expressed on T cells usually leads to a negative regulation of T cell proliferation [51]. A possible explanation for this unexpected finding could be that the gene expression level of *PDCD1LG2* is downregulated by PDL2-independent immunosuppressive mechanisms or that binding of PDL2 to PD-1 induces a negative feedback loop in MSCs i.e. downregulation of *PDCD1LG2*.

ORA showed an upregulation of the SMAD protein signal transduction in 3D culture primed MSCs, which is typically initiated by the binding of transforming growth factor-beta (TGFβ) ligands to their respective receptors. Bone morphogenic proteins (BMPs) – members of the TGFβ superfamily – were shown to inhibit T cell activation and promote macrophage polarization towards an inflammation-resolving phenotype [52–55]. *BMP2* and *BMP6* were highly upregulated in 3D culture primed MSCs and among the top 10 immunomodulation-associated DEGs (supplementary Table 2). This suggests a potential role of the TGFβ/SMAD signaling pathway in the T cell suppressive mechanism of 3D culture primed MSCs. A previous study suggested the involvement of a specific anti-inflammatory mechanism in 3D culture primed MSCs. The proposed mechanism operates at the post-transcriptional level by destabilizing the mRNAs encoding pro-inflammatory cytokines [25]. However, further studies are needed to unravel the immunomodulatory mechanisms of 3D culture primed MSCs.

Moreover, we found an enhanced differentiation potential of 3D culture primed MSCs on transcriptomic level. Angiogenesis (GO:0001525, GO:0045766), cartilage development (GO:0051216), and osteoblast differentiation (GO:0001649) were significantly enriched biological processes in 3D culture primed MSCs. This is in agreement with previous studies [11, 41, 56–58]. Therefore, 3D culture priming might be an interesting tool to simultaneously enhance the immunomodulatory and regenerative potential of MSCs.

In summary, we showed that three MSC priming strategies to increase the immunomodulatory potential result in different immunosuppressive potencies: pro-inflammatory priming induced an almost complete T cell suppression in vitro, 3D culture priming around 40%, and hypoxic priming had no effect. However, priming effects were transient and rapidly lost, yet continuous 3D culture was able to maintain the immunosuppressive potential. The cellular mechanisms leading to T cell suppression were different in pro-inflammatory and 3D culture priming. Pro-inflammatory priming activated inflammatory and immune mechanisms, 3D culture priming induced mechanisms involving the cytoskeleton, cellular signaling, and the cell cycle.

This study had several limitations. The experimental setup of our in vitro functional immunosuppressive potency assay had two major limitations. First, MSCs were co-cultured with PBMCs isolated from a single donor. The activation of T cells via a mixed lymphocyte reaction using PBMCs from multiple donors could have ensured that the assay results reflected more accurately the effects on a representative population. Second, MSC spheroids had to be dissociated prior to the immunosuppressive potency measurements. No suitable in vitro functional assay to measure the immunosuppressive potency of MSC spheroids exists. Therefore, we had to enzymatically digest the spheroids prior to the potency measurements. We decided to strictly adhere to a single in vitro potency assay to ensure comparability of the immunosuppressive potential measurements between the different priming strategies.

The selection of signature genes followed strict criteria including a comparison of potential signature genes with published immunomodulatory potency markers of MSCs. The majority of published immunomodulatory potency markers were identified in studies using pro-inflammatory priming [8, 34, 42]. This possibly introduced a bias into the selection of our signature genes, as we favored the selection of immunomodulatory signature genes that are mainly relevant for pro-inflammatory primed MSCs. This would explain the large number of signature genes that demonstrated a very strong correlation with the T cell suppressive capacity of pro-inflammatory primed MSCs and might explain the absence of signature genes that demonstrated relevant correlations with the T cell suppressive capacity of 3D culture primed MSCs. Nevertheless, it shows that 3D culture priming engages a different mechanism. The assessed signature genes might not reflect the immunosuppressive mechanism of 3D culture primed MSCs, as 3D culture is an emerging priming strategy, and only few potential immunomodulatory potency markers have been identified [12, 42, 59]. . The top 10 immunomodulation-associated DEGs (supplementary Table 2), could serve as unbiased potential immunomodulatory potency markers to bypass the selection bias of immunomodulatory signature genes.

In conclusion, we demonstrated that priming can be used to improve the immunosuppressive potency of MSCs and that translationally relevant in vivo conditions impact the preservation of priming effects. Continuous 3D culture could act as a functionalized formulation, supporting the administration of MSC spheroids for a sustainably improved immunosuppressive potency. Further studies are needed to evaluate the efficacy of 3D culture primed MSCs in pre-clinical and clinical settings.

### Electronic supplementary material

Below is the link to the electronic supplementary material.

Supplementary Material 1

## Data Availability

The RNA sequencing dataset presented in this study can be found in an online repository. The name of the repository and accession number can be found at: https://www.ebi.ac.uk/ena, PRJEB72131.

## Abbreviations

3D: three dimensional
BMP: bone morphogenic protein
BP: biological processes
CCL2: CC motif chemokine ligand 2
CCL5: CC motif chemokine ligand 5
CD274: programmed cell death 1 ligand 1
CD74: major histocompatibility complex class II invariant chain
cDNA: complementary deoxyribonucleic acid
CFSE: Carboxyfluorescein succinimidyl ester
CO_2_: carbon dioxide
CXCL10: CXC motif chemokine ligand 10
CXCL11: CXC motif chemokine ligand 11
CXCL8: CXC motif chemokine ligand 8
CXCL9: CXC motif chemokine ligand 9
DEGs: differentially expressed genes
ELISA: enzyme-linked immunosorbent assay
FDR: false discovery rate
GO: gene ontology
HLA-DRA: major histocompatibility complex class II DR alpha
HMOX1: heme oxygenase 1
ICAM1: intercellular adhesion molecule 1
IDO1: indoleamine 2,3diogygenase 1
IFN-γ: interferon gamma
IL1RN: interleukin 1 receptor antagonist
IL6: interleukin 6
ISCT: International Society of Cell and Gene Therapy
LEP: leptin
LGALS1: galectin 1
LIF: leukemia inhibitory factor
mRNA: messenger ribonucleic acid
MSCs: mesenchymal stromal cells
O_2_: oxygen
ORA: over-representation analysis
PBMCs: peripheral blood mononuclear cells
PBS: phosphate-buffered saline
PCA: principle component analysis
PD-1: programmed cell death protein 1
PDCD1LG2: programmed cell death 1 ligand 2 (gene)
PD-L2: programmed cell death 1 ligand 2 (protein)
PTGS2: prostaglandin-endoperoxide synthase 2
qPCR: quantitative real-time polymerase chain reaction
RNA: ribonucleic acid
TBP: TATA-box binding protein
TGFB1: transforming growth factor beta 1
TNFAIP6: TNF alpha induced protein 6
TNF-α: tumor necrosis factor alpha
