# Supplementary material for: Impacts of priming on distinct immunosuppressive mechanisms of mesenchymal stromal cells under translationally relevant conditions
Source: Stem Cell Res Ther. 2024 Mar 5;15:65. doi: 10.1186/s13287-024-03677-5 (PMC10916130; doi:10.1186/s13287-024-03677-5)
Supplement: Supplementary file 1 — Supplementary Material 1 [file 13287_2024_3677_MOESM1_ESM.docx]

**Supplementary Information**

| **Supplementary Table 1** MSC donor information | | |
| --- | --- | --- |
|  | **Age** | **Sex** |
| Donor 1 | 20 | f |
| Donor 2 | 19 | m |
| Donor 3 | 25 | m |


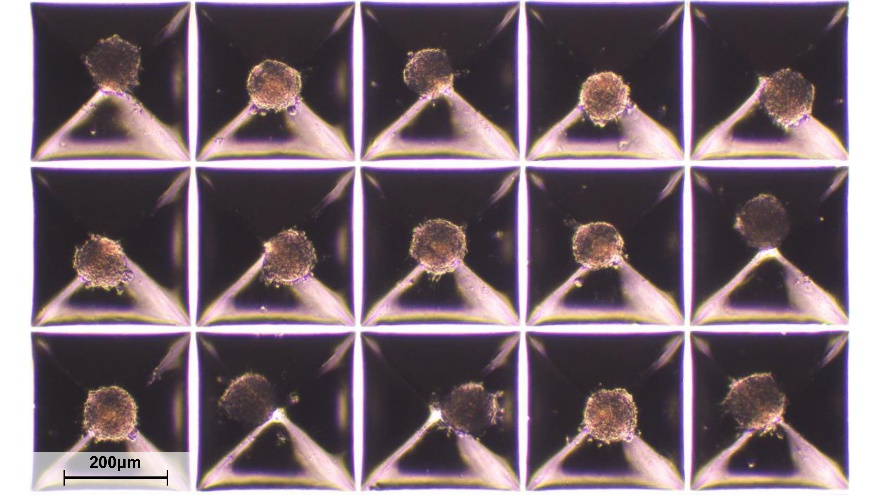

**Supplementary Fig. 1** Representative image of 3D culture primed MSCs in Kugelmeiers SP5D at 48 h post-seeding.

**
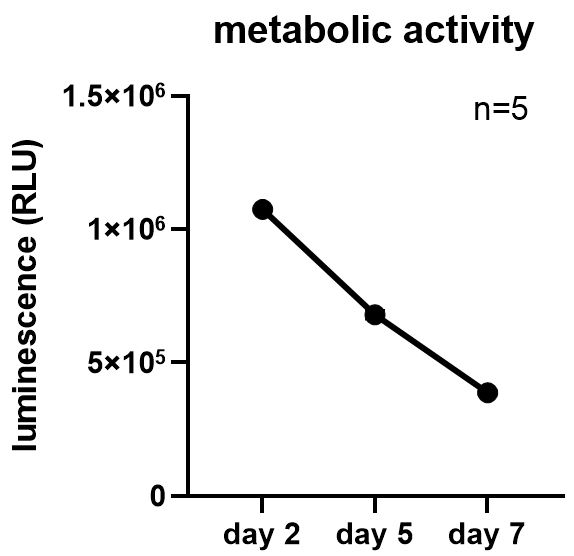

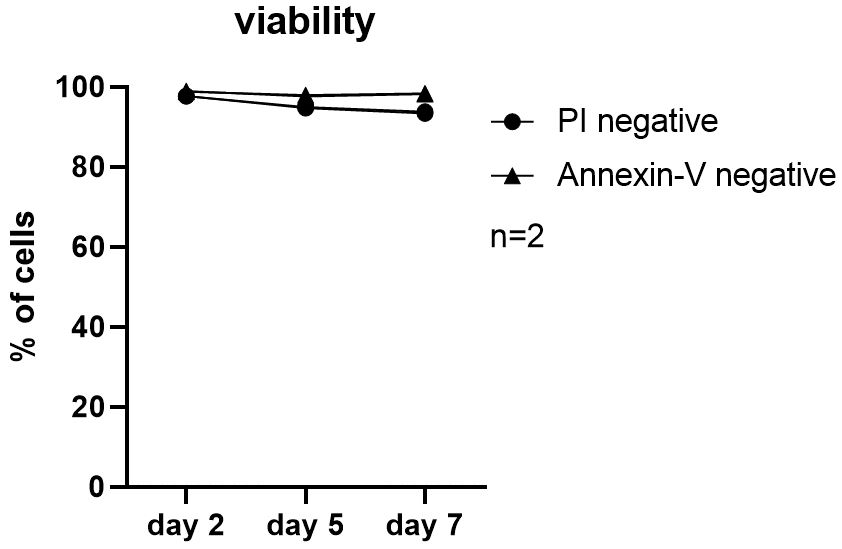
**

**Supplementary Fig. 2** Metabolic activity and viability of MSCs cultured as spheroids over one week. MSCs from one healthy donor were used for assessing the metabolic activity of MSC spheroids. Data points represent averages ± standard deviations of the metabolic activity measurements of five technical replicates. Lines connect the average metabolic activity of each time point. MSC spheroid viability was assessed using MSCs from two healthy donors. Lines connect the average viability of the MSC spheroids for each time point.


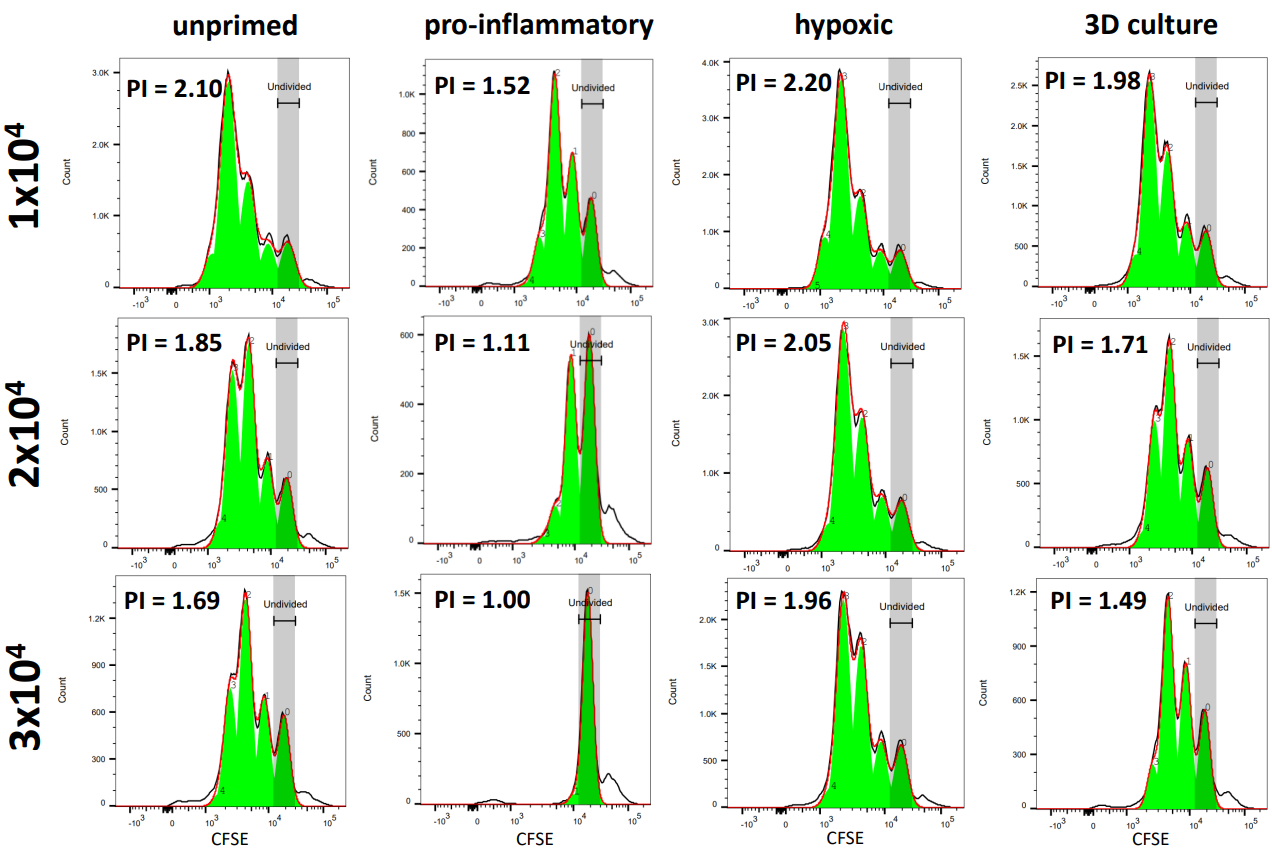

**Supplementary Fig. 3** *Identification of an optimal MSC:PBMC ratio for the in vitro functional potency assay. 1 x 10^4^, 2 x 10^4^, or 3 x 10^4^ primed or unprimed MSCs were co-cultured with 10^5^ CFSE-PBMCs. Their T cell suppressive potencies were assessed directly following priming. MSCs from donor 1 (supplementary Table 1) were used for this titration experiment, and co-cultured with CFSE-PBMCs isolated from an additional healthy donor. PI = proliferation index*

| **Supplementary Table 2** Top 10 immunomodulation-associated DEGs | | | | | | | | |
| --- | --- | --- | --- | --- | --- | --- | --- | --- |
| **pro-inflammatory** | | | **hypoxic** | | | **3D culture** | | |
| **gene** | **log_2_FC** | **FDR** | **gene** | **log_2_FC** | **FDR** | **gene** | **log_2_FC** | **FDR** |
| *CXCL9* | 13.92 | 1.13E-18 | *PIK3R5* | 5.91 | 2.76E-3 | *TNFSF11* | 6.37 | 3.32E-3 |
| *CCL5* | 9.82 | 3.49E-60 | *PTGS2* | 5.31 | 1.23E-51 | *BMP2* | 5.56 | 9.41E-40 |
| *CXCL11* | 8.63 | 1.51E-9 | *APLN* | 5.20 | 4.03E-34 | *BMP6* | 4.20 | 1.75E-11 |
| *HLA-DRB5* | 7.12 | 8.08E-6 | *IL1A* | 4.65 | 1.86E-2 | *NFILZ* | 4.20 | 2.64E-2 |
| *CXCL10* | 6.99 | 4.34E-5 | *CCL28* | 3.93 | 2.01E43 | *PRG4* | 3.94 | 1.09E-13 |
| *HLA-DQA1* | 6.32 | 4.07E-3 | *PRG2* | 3.74 | 1.41E-2 | *LIF* | 3.48 | 1.46E-14 |
| *CD74* | 5.70 | 1.06E-6 | *ACKR3* | 3.50 | 2.22E-5 | *PTGS2* | 3.29 | 8.12E-13 |
| *HLA-DRA* | 5.62 | 5.79E-3 | *PIK3R3* | -5.33 | 5.41E-35 | *THBS1* | -4.37 | <1E-99 |
| *HLA-DRB1* | 5.34 | 1.93E-8 | *HLA-DRA* | -4.00 | 7.99E-9 | *PTX3* | -3.49 | 2.72E-21 |
| *IL-32* | 5.34 | 2.24E-16 | *TLR3* | -3.67 | 1.22E-14 | *JCHAIN* | -3.36 | 3.49E-5 |

| **Supplementary Table 3** Primers used for qPCR amplification | | |
| --- | --- | --- |
| **Gene** | **Forward sequence (5'-3')** | **Reverse sequence (5'-3')** |
| CCL5 (RANTES) | CCTGCTGCTTTGCCTACATTGC | ACACACTTGGCGGTTCTTTCGG |
| CXCL9 (MIG) | CCAGTAGTGAGAAAGGGTCGC | AGGGCTTGGGGCAAATTGTT |
| CXCL10 (IP10) | GGTGAGAAGAGATGTCTGAATCC | GTCCATCCTTGGAAGCACTGCA |
| CXCL11 (I-TAC) | AAGGACAACGATGCCTAAATCCC | CAGATGCCCTTTTCCAGGACTTC |
| CXCL8 (IL8) | GAGAGTGATTGAGAGTGGACCAC | CACAACCCTCTGCACCCAGTTT |
| IL6 | AGACAGCCACTCACCTCTTCAG | TTCTGCCAGTGCCTCTTTGCTG |
| CD74 (HLA-DR) | GATGACCAGCGCGACCTTATC | GTGACTGTCAGTTTGTCCAGC |
| PTGS2 (COX-2) | CGGTGAAACTCTGGCTAGACAG | GCAAACCGTAGATGCTCAGGGA |
| LIF | CCAACGTGACGGACTTCCC | TACACGACTATGCGGTACAGC |
| IL1RN | CATTGAGCCTCATGCTCTGTT | CGCTGTCTGAGCGGATGAA |
| CD274 (PD-L1) | TGCCGACTACAAGCGAATTACTG | CTGCTTGTCCAGATGACTTCGG |
| PDCD1LG2 (PD-L2) | ATTGCAGCTTCACCAGATAGC | AAAGTTGCATTCCAGGGTCAC |
| IDO1 | TCATCTCACAGACCACAAGTCA | GCAAGACCTTACGGACATCTCC |
| CCL2 (MCP1) | CAGCCAGATGCAATCAATGCC | TGGAATCCTGAACCCACTTCT |
| TNFAIP6 (TSG6) | TCACCTACGCAGAAGCTAAGGC | TCCAACTCTGCCCTTAGCCATC |
| LEP | GCTGTGCCCATCCAAAAAGTCC | CCCAGGAATGAAGTCCAAACCG |
| HMOX1 | CCAGGCAGAGAATGCTGAGTTC | AAGACTGGGCTCTCCTTGTTGC |
| LGALS1 | TCGCCAGCAACCTGAATCTC | GCACGAAGCTCTTAGCGTCA |
| TGFB1 | TACCTGAACCCGTGTTGCTCTC | GTTGCTGAGGTATCGCCAGGAA |
| ICAM1 | AGCGGCTGACGTGTGCAGTAAT | TCTGAGACCTCTGGCTTCGTCA |
| TBP | TGTATCCACAGTGAATCTTGGTTG | GGTTCGTGGCTCTCTTATCCTC |

CCL5 (C‑C motif chemokine ligand 5), CXCL9 (C‑X‑C motif chemokine ligand 9), CXCL10 (C‑X‑C motif chemokine ligand 10), CXCL11 (C‑X‑C motif chemokine ligand 11), CXCL8 (C‑X‑C motif chemokine ligand 8), IL6 (interleukin 6), CD74 (major histocompatibility complex class II invariant chain), PTGS2 (prostaglandin-endoperoxide synthase 2), LIF (leukemia inhibitory factor), IL1RN (interleukin 1 receptor antagonist), CD274 (programmed cell death 1 ligand 1), PDCD1LG2 (programmed cell death 1 ligand 2), IDO1 (indoleamine 2,3‑diogygenase 1), CCL2 (C‑C motif chemokine ligand 2), TNFAIP6 (TNF alpha induced protein 6), LEP (leptin), HMOX1 (heme oxygenase 1), LGALS1 (galectin 1), TGFB1 (transforming growth factor beta 1), ICAM1 (intercellular adhesion molecule 1), TBP (TATA-box binding protein)


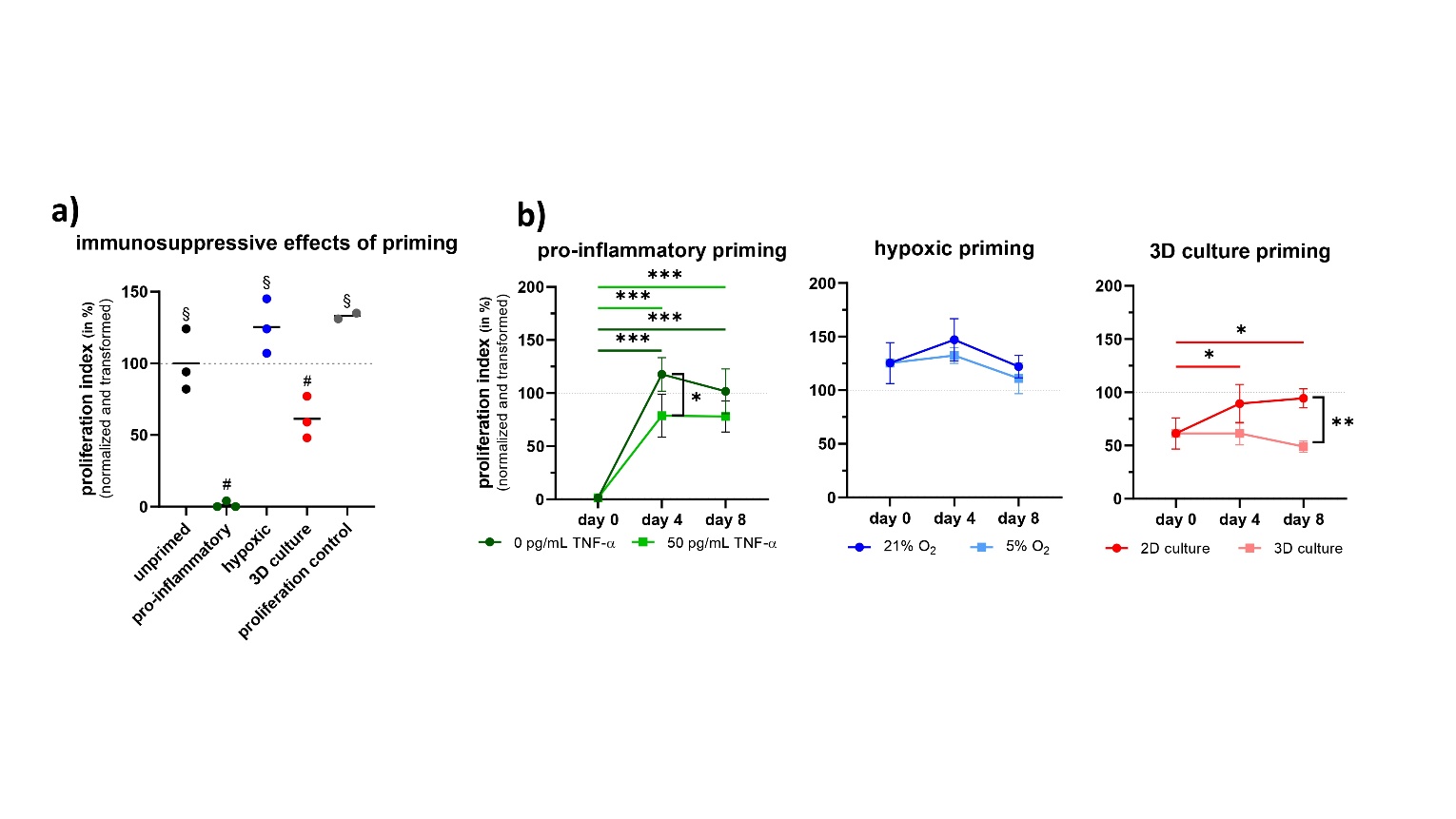


**Supplementary Fig. 4** CD4^+^ T‑helper cells: The effects of each priming strategy on the immunosuppressive potential of MSCs were measured by an in vitro functional potency assay following 48 h of priming. A normalized transformed proliferation index of 0% corresponds to the complete suppression of T cell proliferation. A value of 100% indicates that the T cells proliferated equivalent to T cells co-cultured with unprimed MSCs (grey dashed horizontal lines). **a)** Different priming strategies enhance the T cell suppressive capacity of MSCs. **b)** priming effects fade over time but are partially preserved by translationally relevant conditions. The proliferation control represents the proliferation of T cells in the absence of co-cultured MSCs. Data points represent averages ± standard deviations of the MSCs from three healthy donors. # indicate significant differences (p < 0.001, except unprimed vs. 3D culture primed p = 0.03) against all other conditions. § indicate significant differences (p < 0.001) against pro-inflammatory and 3D culture priming. not significant: p > 0.05; *: p ≤ 0.05; **: p ≤ 0.01; ***: p ≤ 0.001


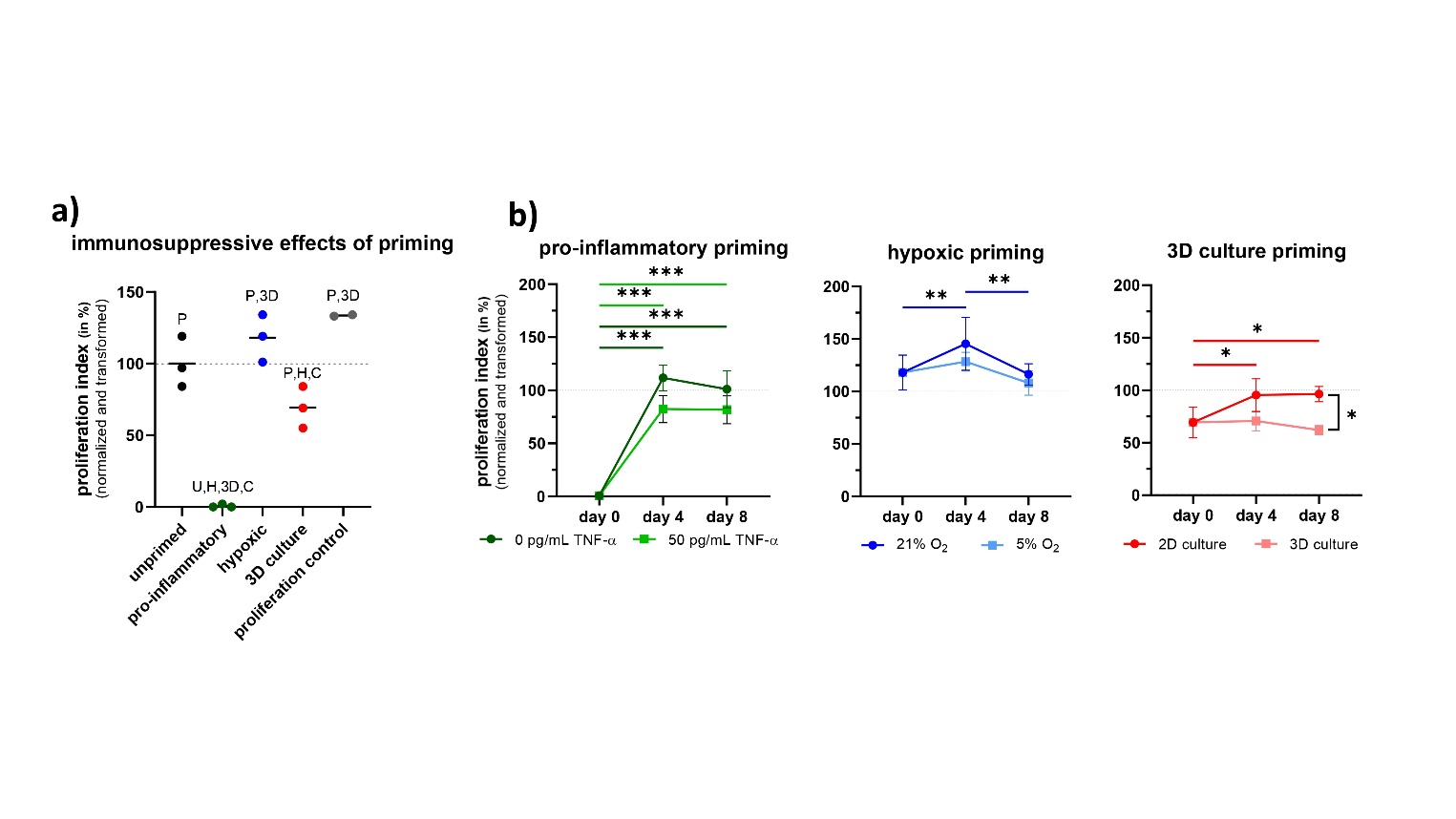


**Supplementary Fig. 5** CD8^+^ cytotoxic T cells: The effects of each priming strategy on the immunosuppressive potential of MSCs were measured by an in vitro functional potency assay following 48 h of priming. A normalized transformed proliferation index of 0% corresponds to the complete suppression of T cell proliferation. A value of 100% indicates that the T cells proliferated equivalent to T cells co-cultured with unprimed MSCs (grey dashed horizontal lines). **a)** Different priming strategies enhance the T cell suppressive capacity of MSCs. **b)** priming effects fade over time but are partially preserved by translationally relevant conditions. The proliferation control represents the proliferation of T cells in the absence of co-cultured MSCs. Data points represent averages ± standard deviations of the MSCs from three healthy donors. Letters indicate significant differences (p < 0.001, except unprimed vs. 3D culture primed p = 0.03) between conditions. U = unprimed, P = pro-inflammatory, H = hypoxic, 3D = 3D culture, C = proliferation control. not significant: p > 0.05; *: p ≤ 0.05; **: p ≤ 0.01; ***: p ≤ 0.001

| **Supplementary Table 4** shared DEGs: pro-inflammatory and hypoxic primed MSCs | | | | |
| --- | --- | --- | --- | --- |
| ACSL5 | CXCL2 | IFIH1 | PLCL1 | SULF2 |
| ADH1B | CXCL6 | IFIT2 | PLD6 | SYTL3 |
| ADRA2A | DDIT4 | IL11 | PPL | TENM2 |
| ANGPT2 | DDIT4L | IL15RA | PRSS35 | TMEM150C |
| APOB | EFHC2 | JCHAIN | PSMB9 | TNFSF13B |
| APOE | EGR3 | KCND2 | PWWP2B | TP63 |
| APOL1 | EPSTI1 | KCNK15 | QPCT | TRIM14 |
| APOL3 | ESM1 | LRP4 | RCOR2 | TSPAN11 |
| ARMCX5-GPRASP2 | ETV7 | MAF | REPS2 | WARS1 |
| ASPN | FGF7 | MGLL | RIPOR3 | XAF1 |
| BDKRB1 | FMO2 | MGP | RPP25 | ZCCHC2 |
| BST2 | FMO3 | MX2 | RTN4RL1 |  |
| C15orf39 | GJD3 | NCKAP5 | SELENOP |  |
| C15orf48 | GPR88 | NPIPB13 | SEMA6B |  |
| CD74 | H1-0 | NTN1 | SEMA6D |  |
| CFB | H4C14 | OAS2 | SERPINB2 |  |
| CFI | HDAC9 | OASL | SFRP4 |  |
| CHI3L1 | HERC6 | OGN | SLC15A3 |  |
| CIITA | HHIP | OMD | SLC40A1 |  |
| CLCA2 | HLA-DRA | OSCAR | SLC44A3 |  |
| CLDN1 | IBSP | PDE1A | SMAD9 |  |
| COL14A1 | ICAM1 | PDE2A | SNED1 |  |
| COL21A1 | ICAM5 | PDE5A | ST8SIA2 |  |
| CXCL1 | IFI35 | PDGFD | STAT1 |  |
| CXCL12 | IFI44L | PDGFRL | STC2 |  |

| **Supplementary Table 5** shared DEGs: pro-inflammatory and 3D culture primed MSCs | | | | |
| --- | --- | --- | --- | --- |
| ADH1B | CMPK2 | IL11 | NTN1 | SELENOP |
| ADRA2A | CTAGE4 | IL1B | OAS1 | SEMA6B |
| AMIGO2 | CXCL6 | IL4I1 | ODF3B | SERPINB2 |
| ANKRD1 | DCLK1 | IL6 | OGN | SFRP4 |
| APCDD1 | DDIT4L | JCHAIN | OMD | SLC40A1 |
| APOB | DLX2 | KCND2 | PDCD1LG2 | SYTL2 |
| APOE | FGF7 | KCNK15 | PDE2A | SYTL3 |
| APOL1 | FMO2 | LEPR | PDE5A | TENM2 |
| ASPN | FMO3 | LIF | PLCL1 | TFPI2 |
| BDKRB1 | GDF15 | MGP | QRICH2 | TMEM132A |
| C15orf48 | GPC4 | NDNF | RGS4 | TSPAN11 |
| CFI | HLA-DRB1 | NPIPB13 | RPP25 | TYMP |
| CHI3L1 | ICAM5 | NPR3 | RSAD2 |  |

| **Supplementary Table 6** shared DEGs: hypoxic and 3D culture primed MSCs | | | | |
| --- | --- | --- | --- | --- |
| ABAT | CPE | GPR68 | NEDD9 | SERPINE1 |
| ADCY8 | CRLF1 | GRAMD2B | NEXN | SFRP4 |
| ADGRG1 | CTXN1 | GREB1 | NGFR | SGMS2 |
| ADH1B | CXCL6 | GRIA3 | NKAIN1 | SHC3 |
| ADM | CYGB | GYG2 | NLGN1 | SLC16A6 |
| ADRA2A | DAAM2 | H2AC18 | NLRP10 | SLC17A7 |
| AKAP5 | DAPK1 | H2AC19 | NMB | SLC22A23 |
| ALPK2 | DCHS1 | H2BC4 | NOTCH3 | SLC22A3 |
| ANGPT1 | DDIT3 | HAPLN1 | NPAS1 | SLC23A3 |
| ANGPTL4 | DDIT4L | HIC2 | NPIPB13 | SLC37A2 |
| APOB | DMD | HMOX1 | NPTX1 | SLC40A1 |
| APOE | DSG2 | HS3ST2 | NR4A2 | SLC46A3 |
| APOL1 | E2F7 | HSF4 | NR4A3 | SLC5A3 |
| ARNT2 | ECM2 | ICAM5 | NTN1 | SLC6A15 |
| ARRDC2 | EFEMP1 | IER3 | NUAK2 | SLC7A14 |
| ARRDC4 | ENPP5 | IGFBP5 | OGN | SMCO4 |
| ASGR1 | ENSG00000279117 | IL11 | OMD | SMIM43 |
| ASPN | ENSG00000279118 | INSYN2A | OSGIN1 | STXBP6 |
| ATP6V0D2 | ENSG00000279773 | ITGA2 | P2RY11 | SYNPO2 |
| B3GALT2 | ENSG00000279881 | JAM2 | PCSK1 | SYT7 |
| B4GALNT2 | ENSG00000280138 | JCHAIN | PDE2A | SYTL3 |
| BAIAP2L1 | ENSG00000280351 | KCNC3 | PDE4C | TC2N |
| BCL11B | ENSG00000284540 | KCND2 | PDE5A | TENM2 |
| BCL2L11 | ERMN | KCNK15 | PGF | TENT5B |
| BDKRB1 | ESCO2 | KCNK2 | PHLDA1 | TGFB3 |
| BMP2 | ETV4 | KCNS1 | PLAAT4 | THBD |
| C11orf87 | FCGBP | KCTD4 | PLCL1 | THBS1 |
| C12orf56 | FER1L6 | KDM7A | PLEKHG4B | TICRR |
| C15orf48 | FGD4 | KIRREL3 | PLIN2 | TLL1 |
| C1orf115 | FGF7 | KLRD1 | PLPP2 | TLR3 |
| C1orf198 | FHIP1A | KRT19 | PODN | TM4SF19 |
| C4orf47 | FILIP1L | KRTAP1-1 | PPFIA3 | TMEM240 |
| CA9 | FKBP5 | KSR1 | PSAT1 | TMEM26 |
| CAMK2N1 | FMO2 | KY | PTGES | TNFAIP6 |
| CAPS | FMO3 | LACC1 | PTGS2 | TNFRSF11B |
| CASP1 | FMO4 | LEF1 | PTPRN | TPBGL |
| CCN2 | FOXQ1 | LMOD1 | PTPRQ | TPD52L1 |
| CD200 | FRY | LOX | RANBP3L | TRIM9 |
| CDKN2B | FZD5 | LRRC66 | RASEF | TSPAN11 |
| CEMIP | FZD8 | LSAMP | RCAN2 | UAP1L1 |
| CFI | GALNT18 | MALL | RIMS1 | VLDLR |
| CH25H | GBP1 | MARCHF4 | RNF144A | VSTM4 |
| CHI3L1 | GDF5 | MCHR1 | ROS1 | WNK4 |
| CHL1 | GDF6 | MCM10 | RPP25 | WNT7B |
| CLK1 | GEM | MEGF6 | SCRG1 | ZNF423 |
| CMKLR1 | GFRA1 | MEOX2 | SELENOP |  |
| COL24A1 | GOLGA8T | MGP | SEMA6B |  |
| CPA4 | GPR35 | MYBL1 | SERPINB2 |  |

| **Supplementary Table 7** shared DEGs: pro-inflammatory and hypoxic and 3D culture primed MSCs | | | | |
| --- | --- | --- | --- | --- |
| ADH1B | CFI | IL11 | OMD | SFRP4 |
| ADRA2A | CHI3L1 | JCHAIN | PDE2A | SLC40A1 |
| APOB | CXCL6 | KCND2 | PDE5A | SYTL3 |
| APOE | DDIT4L | KCNK15 | PLCL1 | TENM2 |
| APOL1 | FGF7 | MGP | RPP25 | TSPAN11 |
| ASPN | FMO2 | NPIPB13 | SELENOP |  |
| BDKRB1 | FMO3 | NTN1 | SEMA6B |  |
| C15orf48 | ICAM5 | OGN | SERPINB2 |  |


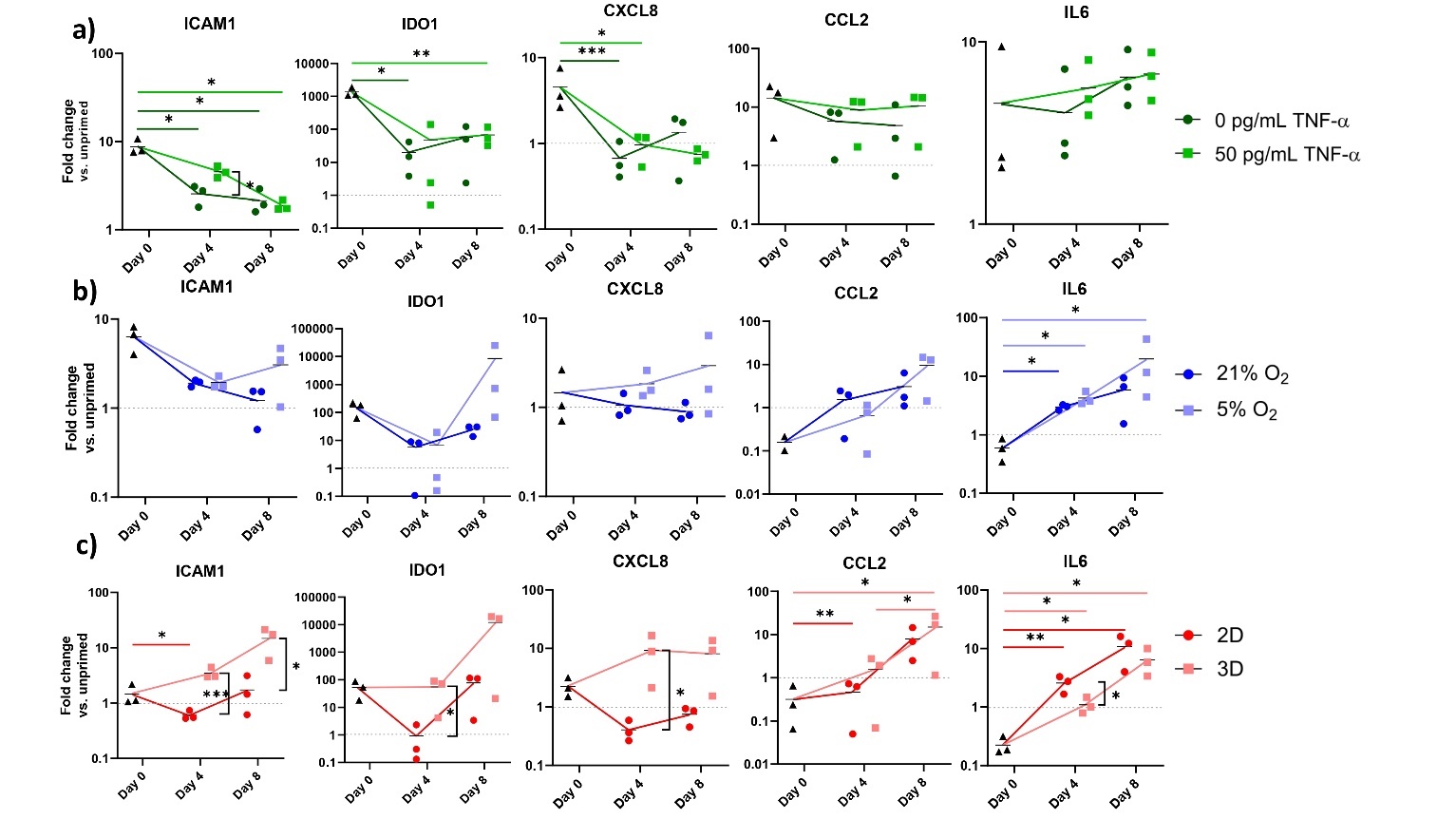


**Supplementary Fig. 6** Fading of frequently used immunomodulatory potency markers. The effects of each priming approach on the transcriptome of MSCs were semi-quantified by qPCR at three time points: day 0 (directly following 48 h of priming), day 4, and day 8. Fold changes are shown and represent the gene expression levels of primed MSCs from three healthy donors compared to the gene expression levels of unprimed MSCs at time point day 0. Lines connect the mean fold changes of the three healthy donors. The significance levels for the gene expression differences between each time point and condition, compared to the gene expression directly after priming at day 0 are shown. Additionally, for each time point separately, significant gene expression differences between primed MSCs cultured under standard conditions and primed MSCs cultured under translationally relevant conditions are highlighted by significance indicators (not significant: p > 0.05; *: p ≤ 0.05; **: p ≤ 0.01; ***: p ≤ 0.001).
